# A Glucose-Responsive CeO₂@GOx Nanozyme Embedded in Chitosan/PVA Hydrogel for Accelerated Diabetic Wound Healing: from Molecular Simulations to *In Vivo* Validation

**DOI:** 10.7150/ntno.132646

**Published:** 2026-03-17

**Authors:** Lidia Grace Naomi, Dino Pati Putra, Rangga Adhi Prastika, Azizah Mirza Kautsari, Dewi Sintawati Try Sutrisno, Suhailah Hayaza, Melisa Intan Barliana, Windri Handayani, Nik Ahmad Nizam Nik Malek, Inna Syafarina

**Affiliations:** 1 Nanotechnology Engineering, Faculty of Advanced Technology and Multidiscipline, Universitas Airlangga, Surabaya 60115, Indonesia.; 2 Airlangga Functional Nanomaterials Research Group, Faculty of Advanced Technology and Multidiscipline, Universitas Airlangga, Surabaya 60115, Indonesia.; 3 Department of Biological Pharmacy, Faculty of Pharmacy, Universitas Padjadjaran, Sumedang, Indonesia.; 4 Department of Biology, Faculty of Mathematics and Natural Sciences, Universitas Indonesia, Indonesia.; 5 Center of Excellence for Pharmaceutical Care Innovation, Faculty of Pharmacy, Universitas Padjadjaran, Sumedang, Indonesia.; 6 Center for Sustainable Nanomaterials (CSNano), Ibnu Sina Institute for Scientific and Industrial Research (ISI- ISIR), Universiti Teknologi Malaysia, 81310 Johor, Malaysia.; 7 Research Center for Computing, National Research and Innovation Agency, Jl. Raya Jakarta Bogor KM 46, Cibinong, Indonesia.

**Keywords:** Diabetes, Cerium nanozyme, Chitosan, hydrogel, wound healing

## Abstract

Diabetes prevalence in Indonesia reached 19.465 million patients in 2021 and is predicted to increase to 40.7 million cases by 2045, with 15-25% of diabetic patients at risk of diabetic wounds and 15-30% threatened with amputation. This study aims to determine the optimal concentration of cerium oxide nanoparticles functionalized with glucose oxidase (CeO_2_@GOx) nanozymes for the treatment of diabetic wounds. CeO_2_@GOx was incorporated into chitosan/poly(vinyl alcohol) (CS/PVA) hydrogel composites via a freeze-thawing method and systematically evaluated through physicochemical characterization, enzymatic assays, *in silico* analysis, antibacterial *in vitro* and *in vivo* wound healing studies. Molecular dynamics simulations (200 ns) were employed to investigate the dynamic stability of the flavin adenine dinucleotide cofactor within the GOx active site (FAD@GOx), particularly its structural persistence in the presence of Ce atom, CS, and PVA. Complementary density functional theory (DFT) calculations were independently performed on Ce₄O₈ clusters, chitosan, and poly(vinyl alcohol) (PVA) to examine their electronic properties and noncovalent interaction characteristics. UV-Vis analysis revealed increased absorbance at 298 nm with increasing CeO_2_@GOx concentrations, while functional group analysis confirmed the presence of hydroxyl, amine, carbonyl, and cerium-related features. The hydrogel exhibited a uniform particle size distribution (PDI = 0.1703) and a degradation rate of 90.334%. Among the tested formulations, the CeO_2_@GOx 4:1 (^w^/_w_) hydrogel exhibited the highest TMB oxidation rate and catalase-like activity and demonstrated optimal diabetic wound-healing performance *in vivo* within 7 days.

## Introduction

Diabetes mellitus is defined as a condition where the body experiences metabolic abnormalities so that it is unable to produce insulin or insulin resistance 1-3. Insulin is a hormone that balances blood glucose level. When the body lacks insulin, blood glucose levels will increase and interfere with the body's metabolism. Fasting blood glucose levels in people with diabetes can reach 125 mg/dL and above, with normal levels around 70-100 mg/dL and below 4. Various health problems can arise due to diabetes, one of which is the risk of diabetic ulcer. Elevated blood glucose level can cause damage to tissues and blood vessels, potentially resulting in recurrent wounds. Consequently, the impaired circulation compromises the wound healing process, making it significantly more challenging for diabetic subjects to recover from such injuries 5,6.

In 2021, according to data from the International Diabetes Federation (IDF), Indonesia ranked seventh in the world for diabetes prevalence with 19.465 million people with diabetes. In addition, IDF also predicts that in 2045 diabetes will occupy the seventh position of the disease that causes death and the prevalence of diabetes is estimated to increase to reach 40.7 million people 7. Diabetic wound complications are not uncommon in people with diabetes, 15-25% of diabetic patients experience diabetic wounds and with the increase in diabetes cases each year, diabetic wound cases will also increase 8. About 15-30% of diabetic wound cases are threatened with amputation and various other life-threatening effects 9,10. Diabetic wound management is challenging because wounds are more sensitive to infection, which is prevented by the administration of antimicrobial drugs either topically or by injection. However, the administration of antimicrobial drugs has not shown optimal results in protecting wounds from infection. Additionally, there is an urgency to develop a therapeutic agent that not only protect diabetic wounds from possible infection, but also reduce oxidative stress to accelerate wound healing.

Based on previous research, cerium oxide nanoparticles are able to mimic the concept of enzymatic activities (nanozymes), starting from superoxide dismutase (SOD), peroxidase, and catalase (CAT) enzymes that effectively break down excess reactive oxygen species (ROS) 11. Glucose oxidase (GOx) can convert glucose and change it into gluconic acid and produce a by-product in the form of hydrogen peroxide (H_2_O_2_), so that glucose concentration is regulated 12,13. This catalytic process is mediated by flavin adenine dinucleotide (FAD), which serves as the redox-active cofactor at the active site of GOx and plays a crucial role in electron transfer during glucose oxidation 14-16. Previous studies have reported that glucose-triggered cascade reactions represent an emerging therapeutic strategy for wound healing, in which GOx-generated H₂O₂ can be further regulated or decomposed by nanozymes to maintain a balanced wound microenvironment 17. Then the hydrogel composite is utilized as a topical application of the material on the wound. The use of chitosan as a material for making hydrogels is being widely researched, because the physicochemical properties of hydrogels can be designed to be responsive to certain external stimuli. In hydrogel fabrication, polyvinyl alcohol (PVA) is also used, which has a water absorption capacity 5-10 times its weight 18.

From the description of the benefits of each component above, this research utilizes cerium oxide nanoparticles functionalized with glucose oxidase (CeO_2_@GOx) in chitosan and PVA-based hydrogel composites (CS/PVA) as diabetic wound healing agents 19,20. The characterization carried out includes the spectrum absorbance using Ultraviolet-Visible spectrophotometer (UV-Vis), functional group characterization using Fourier Transform Infrared Spectroscopy (FT-IR), Particle Size Analyzer (PSA) for the dispersity and stability test, hydrogel degradation test, enzymatic peroxidase and catalase tests, *in silico* analysis, antibacterial *in vitro* assay, and *in vivo* tests. This research will underpin an accessible yet effective treatment for diabetic wounds that supports Sustainable Development Goals (SDGs) point 3 of healthy living and well-being.

## Materials and Methods

### Materials

The research materials used include cerium (III) nitrate hexahydrate (Ce(NO_3_)_3._6H_2_O) (Merck, Germany)*,* citric acid (C_6_H_10_O_8_) (Merck), glucose oxide (GOx) from *Aspergillus Niger* (Chatson Jaya Lab, Indonesia), ethylene diamine tetraacetic acid (EDTA) (Merck), aquadest (H_2_O), ammonium hydroxide (NH_4_OH) (Merck, Germany), ethanol (C_2_H_6_O), (3-Aminopropyl)triethoxysilane (C_9_H_23_NO_3_Si) (Sigma Aldrich), phosphate buffer saline (PBS) pH 7,4 (Nitra Kimia), chitosan (CS) (Sigma Aldrich, USA), polyvinyl alcohol (PVA) (Sigma Aldrich, USA), acetic acid (CH_3_COOH) (Supelco, Germany), Tetraethyl orthosilicate (TEOS) (Sigma Aldrich), aquadest, hydrogen peroxide (H_2_O_2_), tetramethylbenzidine (TMB) (Merck), glutaraldehyde (C_5_H_8_O_2_) (Merck), citrate buffer, alcohol, ketamine (C_13_H_16_ClNO), and streptozotocin (STZ) (Chem Cruz, USA).

### Methods of functionalization of CeO_2_@GOx CS/PVA

#### Density Functional Theory (DFT)

Density functional theory (DFT) calculations using ORCA version 6.0.1 were employed to investigate the electronic structure and noncovalent interactions features of representative molecular models. Geometry optimizations were performed using the hybrid PBE0 exchange-correlation functional together with the def2-TZVP basis set 21,22. Long-range dispersion effects were accounted for by applying Grimme's D3 correction with Becke-Johnson damping (D3BJ) 22,23. To improve computational efficiency, the resolution of identity approximation within the RIJCOSX scheme was adopted in conjunction with the def2/J auxiliary basis set 22. Tight self-consistent field (TightSCF) convergence criteria were applied throughout all calculations 24,25. CS and PVA were represented by short molecular segments to describe their local electronic environments. Based on previous theoretical studies reporting stable Ce-O coordination in Ce₄O₈ cluster 26. In this research, Ce₄O₈ cluster was adopted to represent the local electronic environment of cerium oxide.

#### Molecular dynamics simulation

The 3D structure of glucose oxidase (GOx) was obtained from the Protein Data Bank (PDB ID: 1GAL). Protein modeling and reconstruction of missing residues were performed using the SWISS-MODEL (https://swissmodel.expasy.org/) (Fig. S1 and Fig. S2) 27. The protein was identified using the PrankWeb (https://prankweb.cz/) to obtain active site of the protein (Table S1) 28. Molecular docking was employed solely to generate initial protein-ligand conformations for subsequent molecular dynamics simulations, with the docking region defined around the FAD binding site. Docking calculations were performed using the AutoDock Vina package 29, while Ce atom, chitosan and polyviniyl alcohol docked were positioned within the predicted active site region. The docking grid box was defined with center coordinates (X: 47.9287 Å, Y: 15.4710 Å, Z: 58.6315 Å) and box dimensions (X: 14.479 Å, Y: 47.8644 Å, Z: 22.9982 Å). Docking poses were analyzed and visualized using UCSF Chimera software 30. Molecular dynamics simulations were performed using the GROMACS 2023.02 package based on the CHARMM Generalized Force Field (CGENFF) 31. The FAD@GOx complex obtained from the docking stage was used as the initial structure. The system was described using addition Ce atom parameter for CHARMM-All36 forcefield 32, with appropriate parameters assigned to the protein and the FAD cofactor. Energy minimization was conducted to remove steric clashes, followed by equilibration under constant volume (NVT) and constant pressure (NPT) conditions at 310 K and 1 atm for a a total of 4 ns. A production MD simulation was then carried out for 200 ns under periodic boundary conditions. Binding free energy calculations were carried out using the Molecular Mechanics/Generalized Born Surface Area (MM-GBSA) approach implemented in the *cpptraj* and *MMPBSA.py* 33. The binding free energy (∆*G_bind_*) was evaluated by combining the gas-phase interaction energy (∆*G_gas_*) and the solvation free energy contributions (∆*G_solv_*). As described in Eqn. (1), the (∆*G_gas_*) represents the interaction energy between the ligand and the receptor in the absence of solvent, obtained from the difference between the complex and the separated components. The (∆*G_solv_*) was calculated as shown in Eqn. (2). Furthermore, the solvation contribution was decomposed into electrostatic (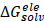) and nonpolar components (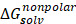), as defined in Eqn. (3).


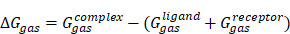

(1)


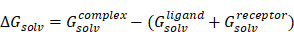

(2)


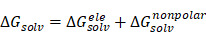

(3)

#### Synthesis of CeO_2_ NPs

The synthesis of CeO_2_ NPs was performed by the precipitation method at 27°C. First, 10 mL of 1% EDTA solution, 10 mL solution of 1% C_6_H_10_O_8_ was added into 10 mL of Ce(NO_3_)_3._6H_2_O 0.8 M. Then, add 50 mL of 0.8 M NH_4_OH dropwise and stirred at 450 rpm. After 24 hours of mixing, the samples were transferred into falcon tubes and left at room temperature for 2 days. The sample was centrifuged at 4,000 rpm for 20 minutes and the precipitate was collected. Then, the precipitate was washed using distilled water twice and ethanol once, then dried for 15 hours at 60°C. In the final stage, the sample was crushed to a fine yellow powder of CeO_2_ NPs.

#### Synthesis and immobilization of CeO_2_@GOx

The formation stage of CeO_2_@GOx begins with CeO_2_ NPs re-dispersed in 50 mL ethanol via sonication for 15 minutes. Then, 200 μL of 3-(aminopropyl)triethoxysilane was added, stirred at room temperature overnight. The precipitate was washed with 3x4 mL of ethanol and dried in air at room temperature. The amino-functionalized CeO_2_ NPs were then washed with 4x4 mL PBS and re-suspended in 5 mL PBS solution. On the other hand, GOx was dissolved into 2 mL of PBS solution pH 7.4 and incorporated into the CeO_2_ NPs solution. The reaction mixture was gently stirred overnight. The CeO_2_@GOx solution was washed with 4 times 4 PBS solution pH 7.4. The solution was resuspended in 5 mL of PBS solution pH 7.4.

### Synthesis of CeO_2_@GOx and incorporation into CS/PVA hydrogel composites

CS/PVA hydrogel was synthesized by first dissolving CS in 1% (^w^/_v_) acetic acid at room temperature for 24 h under constant stirring. In parallel, PVA was dissolved in distilled water (10% ^w^/_v_) at 90-99°C until a homogeneous solution was obtained. Subsequently, the PVA solution was added to the CS solution, followed by the addition of 2 mL of TEOS. The mixture was stirred at 70°C for 3 h until homogeneous. After cooling to room temperature, the solution was ultrasonicated for 5 min without temperature control. Separately, the CeO_2_@GOx solution was suspended in PBS and sonicated for 10 min. The CeO_2_@GOx suspension was then added dropwise into the CS/PVA solution under continuous stirring for 2 h. The resulting mixture was cast into a petri dish and allowed to rest at room temperature for 30 min. The petri dish was covered with aluminium foil and left undisturbed for 24 h. The formed hydrogel was then transferred into a microtube and subjected to a freeze-thaw process at -20°C until completely frozen. After freezing, the hydrogel was allowed to thaw at room temperature, and its structure was subsequently observed 34,35.

### Characterization of CeO_2_@GOx CS/PVA

#### Spectrophotometric evaluation using UV-Vis

Ultraviolet-Visible (UV-Vis) is a spectroscopic technique commonly used for the measurement of light absorbance at various wavelengths that are still in the ultraviolet (UV) range (190-400 nm) and visible (Vis) range (400-800 nm) derived from the electromagnetic spectrum 36. The UV-Vis test was used to determine the responsiveness of the nanozyme sample to the absorbance of CeO_2_ NPs and GOx respectively in the CeO_2_@GOx sample variation using the GENESYS 180 UV-Vis Spectrophotometer, where all samples were in liquid form for UV-Vis analysis. In addition, this test is also used to determine the absorbance intensity when there is the addition of CeO_2_ NPs in CeO_2_@GOx 37,38.

#### Functional group characterization using FT-IR

Functional group characterization was carried out using fourier transform infrared (FT-IR) Spectroscopy is a tool to analyze a functional group in a chemical compound 39. The FT-IR test used The PerkinElmer Spectrum™ 3 FT-IR spectrometer with a wave number range from 4000-400 cm^-1^. The samples characterized were in liquid form, including CeO_2_@GOx, CS/PVA, and all variations of the variation of hydrogel samples such as CeO_2_@GOx CS/PVA 1:1, CeO_2_@GOx CS/PVA 2:1, and CeO_2_@GOx CS/PVA 4:1. Prior to measurement, the FT-IR detection area was thoroughly cleaned with ethanol to eliminate residual impurities and minimize background interference during spectra acquisition.

#### Particle size diameter test

The particle size diameter test employs dynamic light scattering (DLS) to assess the dispersion of a system 40,41. Particle size analysis (PSA) typically measures the Brownian motion of individual particles in a liquid to analyze their size. The PSA assay was utilized to evaluate the dispersity of CeO_2_ NPs and CeO_2_@GOx, and to measure their particle size diameter using a BIOBASE BK-802N device, which measures in the range of 1-1000 nm.

#### Degradation test

The degradation test with PBS was performed to simulate the hydrogel's behaviour in the body when used as a diabetic wound dressing 34. The degradation test was carried out by immersing all samples in PBS solution pH 7.4 and left in a room around 25°C for 192 hours or 8 days. To evaluate the physical resistance of the samples in degradation, the samples were weighed every day to measure the percent mass ratio of the initial mass of the samples using PBS. Degradation was carried out under the closest conditions for the interaction between hydrogel and skin in a biological environment 42, so PBS solution was chosen to facilitate this 34. In the calculation of degradation, the following formula of Eqn. (4) was used:


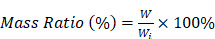

(4)

with W as the mass per day and W_i_ as the initial mass of the sample.

### Enzymatic test of CeO_2_@GOx

The peroxidase and catalase enzymatic assays were performed according to the research references with some special adjustments and modifications 37,43. Catalase is one of the working nanozymes used to degrade H_2_O_2_, an oxidizing agent harmful to the diabetic wound environment 35. Nanocerium shows catalase-like behavior, especially when the Ce^+4^/Ce^+3^ ratio is elevated, as evidenced by methods such as the Amplex Red assay and UV-Vis spectrophotometry, where H_2_O_2_ absorption is tracked at 240 nm. Peroxidases, enzymes typically characterized by the presence of a heme cofactor in their active sites, are prevalent in living organisms; glutathione peroxidase is one notable example 20. Peroxidase and catalase mimetic activities utilize hydrogen peroxide as a substrate and break it down into harmless water and oxygen molecules in catalase activity and/or generate hydroxyl and perhydroxyl radical cascades in peroxidase activity at acidic pH values 43. In enzymatic peroxidase reactions, substrates such as the colorless 3,3',5,5'-tetramethylbenzidine (TMB) undergo oxidation, resulting in the formation of a colored product in the presence of peroxidase 20.

### Antimicrobial assay

An antimicrobial assay was done to analyze the CeO_2_@GOx sample capabilities further to prevent possible wound bacterial infections. The antimicrobial assay was achieved through an inhibition zone test named disc diffusion assay (DDA). Gram-negative bacteria *Escherichia coli* ATCC 25922 (*E. coli*) and Gram-negative bacteria *Staphylococcus aureus* ATCC 25923 (*S. aureus*) were used in this test as the bacterial representative. *Escherichia coli* and *Staphylococcus aureus* were grown in NB (nutrient broth) media and incubated for 24 hours at 37℃. A 100 µl sample of 24-hour-old microorganisms, which had reached an OD = 0.1 in physiological water, was taken and spread on a Petri dish that had been pre-filled with MHA (Mueller Hinton Agar) media. MHA media was used to test antimicrobial samples against the growth of *E. coli* and *S. aureus*. The samples used in this test were in liquid to semi-gel form with the codes CeO_2_@GOx 1:1, 2:1, and 4:1 ^w^/_w_. The samples were taken in a volume of 30 µl and dropped onto a sterile blank disc. The positive control used gentamicin as a commercial antimicrobial agent, and the negative control used aquadest. Then, the Petri dishes were incubated at 37℃ for 24 hours. The diameter of the inhibition zone formed around the disc containing the sample was measured using calipers. The blank disc used had a size of 5 mm 44,45.

### *In vivo* study

*In vivo* tests were performed on male BALB/c strain mice (*Mus musculus*), 4 weeks of age, and body weight 20-30 grams, divided into four different treatment groups, namely the Normal Group (KN) without being given Streptozotocin (STZ), the Negative Group (K-) given STZ, the Positive Group (K+) given STZ and given conventional diabetic wound medication in the form of Betadine ointment, and the Treatment Group (KP) given a hydrogel composite with the best concentration based on characterization and a series of previous tests. STZ was administered at a dose of 30 mg/kg once a day, for five consecutive days based on multiple low-dose diabetes index. After induction, only the K-, K+, and KP groups were confirmed to have developed diabetic conditions and proceeded to treatment, while the KN group was injected with a citrate buffer to ensure all mice were subjected to equivalent stress conditions. After that, visual observation of wound healing was carried out on mice for 7 days 1. The number of mice for 4 groups was adjusted according to Federer's formula, resulting in a minimum of 6 mice in 1 group and a total of 24 mice. The procedure of this study was approved by the Research Ethics Commission, Faculty of Veterinary Medicine, Airlangga University (No. 3.KEH.105.07.2024).

## Results and Discussion

### Density functional theory (DFT) study

**Figure 1:**
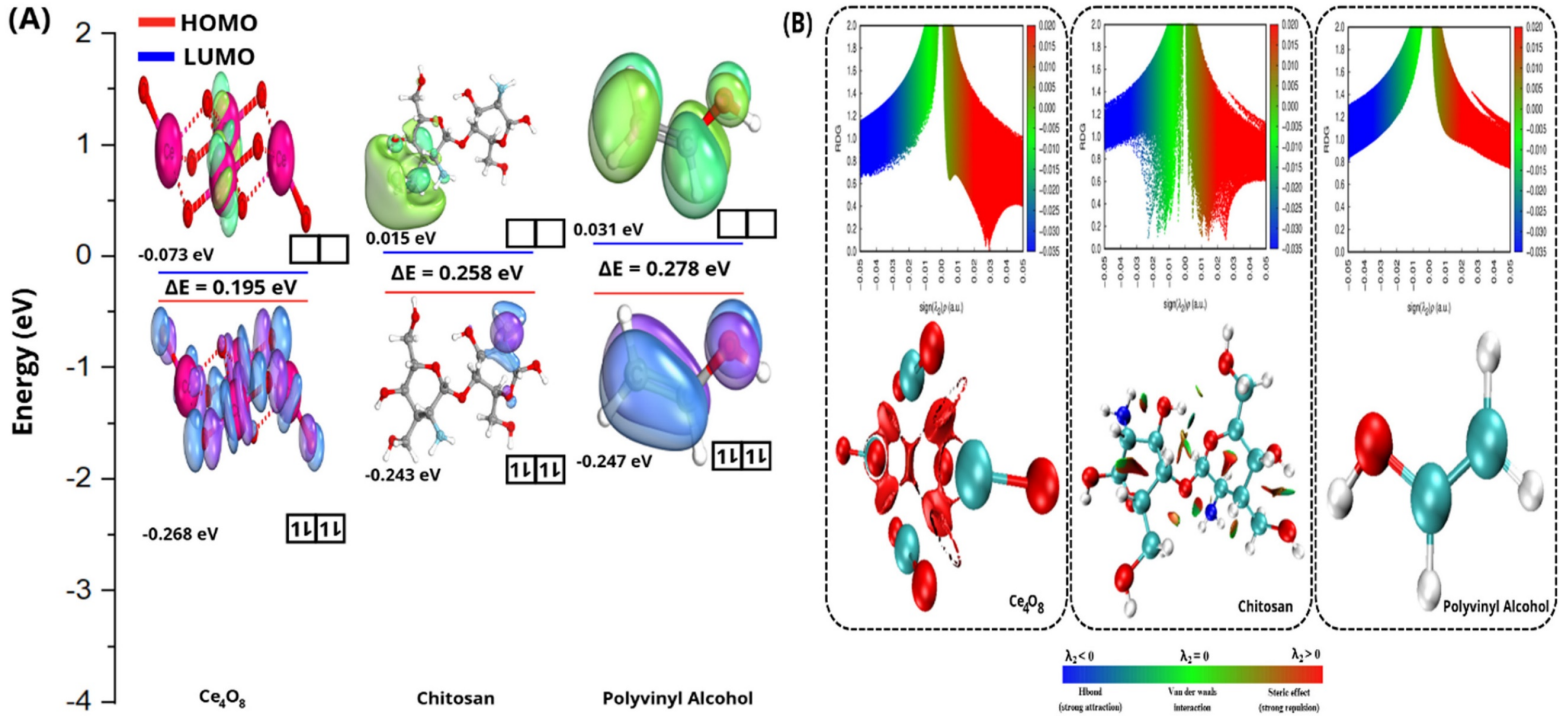
Electronic properties; **(A)** HOMO-LUMO with their energy levels and energy gaps (ΔE), and **(B)** RDG analysis.

The electronic properties of Ce₄O₈ cluster, CS, and PVA were analyzed through HOMO-LUMO and Reduced Density Gradient (RDG) analyses (Figure 1A). The HOMO-LUMO distributions illustrate the electron-donating and electron-accepting regions of each system, while the energy gap (ΔE) reflects their electronic stability and reactivity 46,47. Ce_4_O_8_ exhibits the smallest energy gap, indicating higher electronic reactivity compared to chitosan and PVA. In contrast, chitosan and PVA show relatively larger ΔE values, suggesting more stable electronic structures. In addition, the energies of the HOMO and LUMO were employed to derive global reactivity descriptors, including chemical hardness (η), softness (S), chemical potential (μ), electronegativity (χ), electrophilicity index (ω), and the maximum amount of electronic charge transfer (ΔN_max_). These descriptors provide quantitative insight into the intrinsic electronic behavior of each system and presented in Table 1. The descriptors were calculated using Eqn. (5-10) based on established theoretical formulations reported in previous studies 48-50.


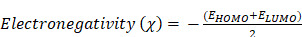

(5)


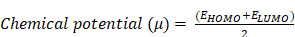

(6)


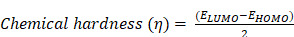

(7)


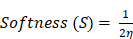

(8)


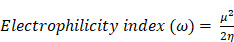

(9)


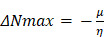

(10)

The reduced density gradient (RDG) analysis was further employed to visualize and characterize the noncovalent interactions present in the Ce₄O₈ cluster, CS, and PVA (Figure 1B). The RDG plots, represented as scatter graphs of RDG versus sign(λ₂)ρ, enable the identification of different types of interactions. Peaks located at negative values of sign(λ₂)ρ correspond to attractive interactions such as hydrogen bonding, while regions around zero indicate van der Waals interactions, and positive values are associated with steric repulsion. Gradient isosurfaces offer an intuitive visualization of weak interactions as spatially extended regions, rather than as isolated pairwise atomic contacts 51. When the reduced density gradient is mapped onto the molecular geometry, continuous surfaces appear, allowing the identification of green regions associated with attractive interactions and red regions corresponding to steric effects 51-53.

### Molecular dynamics simulation study

**Figure 2:**
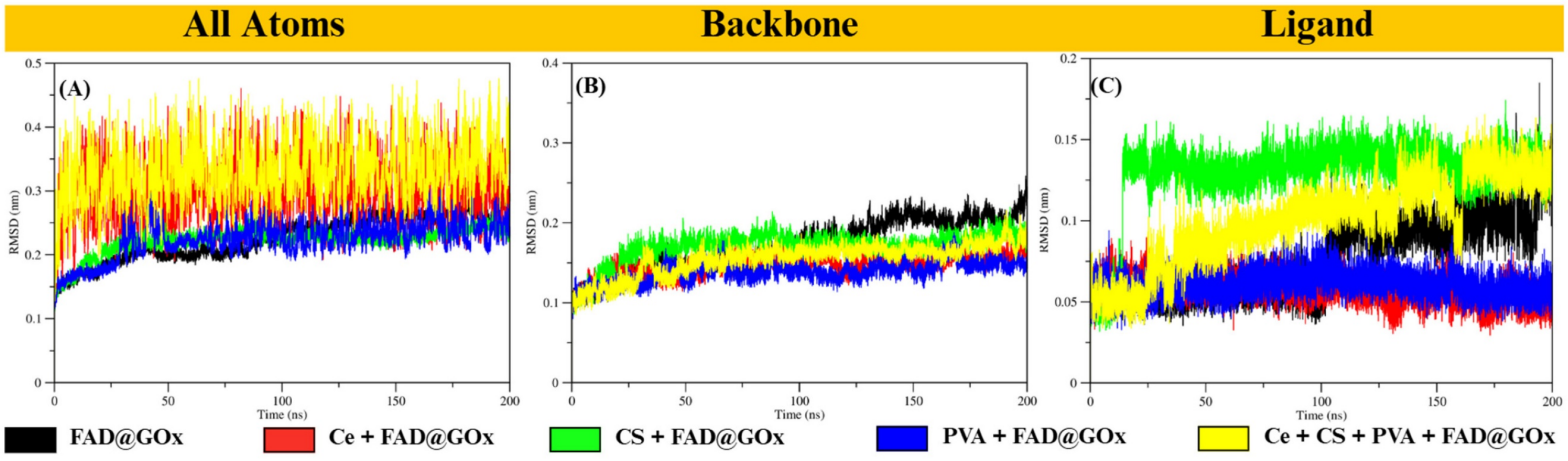
RMSD analysis of FAD@GOx systems under different simulation conditions: **(A)** All atoms RMSD, **(B)** backbone, and **(C)** ligand RMSD with a specific focus on the FAD cofactor relative to the GOx structure.

To evaluate the structural stability of GOx and the behavior of the FAD cofactor under different simulation conditions, molecular dynamics simulations were performed and analyzed using root mean square deviation (RMSD) analysis (Figure 2). The RMSD behavior observed in this study is consistent with previously reported molecular dynamics simulations of the GOx-FAD complex 33. In earlier studies, GOx systems exhibited an initial increase in RMSD followed by stable fluctuations, which was attributed to conformational adjustment during equilibration while preserving the overall protein fold. Similarly, in our simulations, the all-atom RMSD in the Ce + FAD@GOx and Ce + CS + PVA + FAD@GOx systems increases from approximately 0.2 to 0.4 nm before reaching a stable plateau, indicating structural relaxation without significant unfolding. Notably, the RMSD values obtained in this work remain within a comparable or lower range than those reported for GOx-FAD and GOx-FAD-glucose complexes, suggesting that the incorporation of Ce atom, CS, and PVA maintains the global structural stability of the GOx structure. However, analysis of the Ce atom trajectory reveals that, over the 200 ns simulation, Ce tends to migrate away from the GOx surface, particularly in the presence of the CS-PVA matrix (Figure S3). This behavior indicates that Ce does not form persistent direct interactions with the protein, and its influence on GOx is likely indirect, mediated through the surrounding environment rather than strong binding to the active site. Overall, the preserved RMSD profiles confirm that the global fold of GOx remains stable throughout the simulation, while local flexibility-especially in the FAD-binding region is retained. The average RMSD values for all systems are summarized in Table S2.

**Figure 3:**
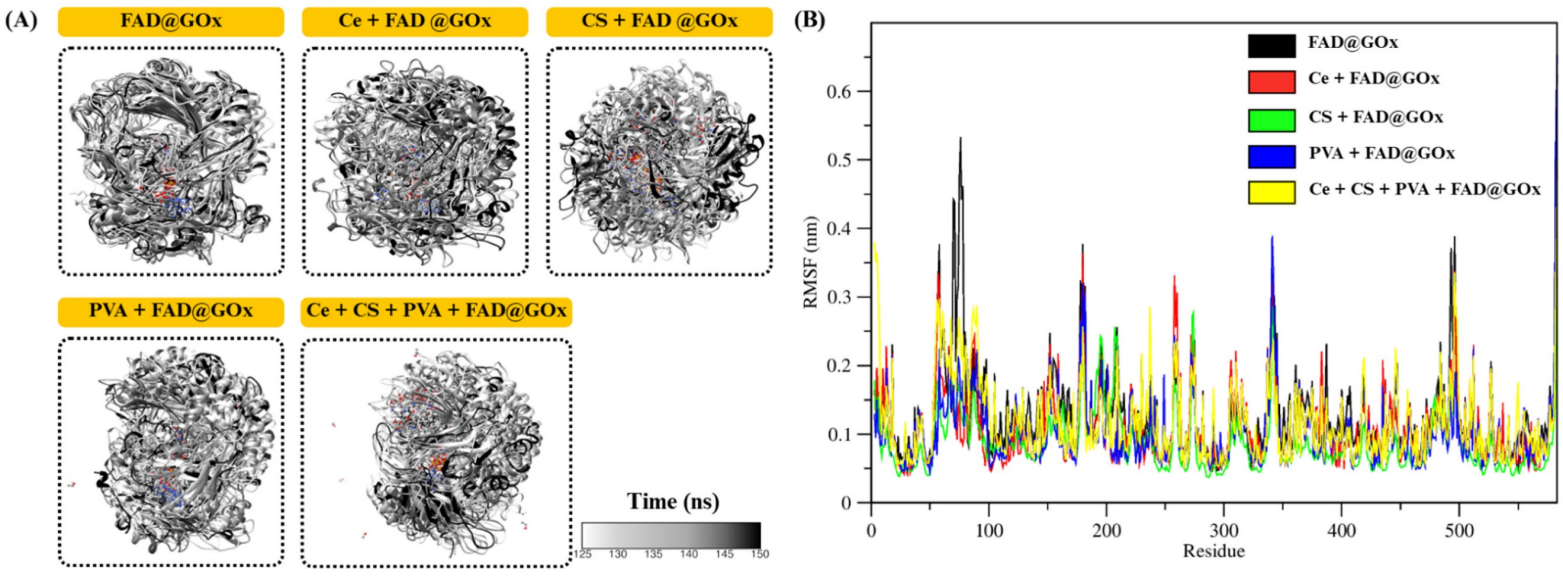
Structural and dynamic properties of FAD@GOx systems; **(A)** Representative structural snapshots at 125-150ns for simulations system, **(B)** RMSF profiles of FAD@GOx residues.

To gain insight into the conformational stability of GOx and the dynamic behavior of its catalytic environment, the structural evolution of the FAD@GOx systems was examined during the production phase of the molecular dynamics simulations 54,55. Particular attention was given to the preservation of the overall protein fold and the integrity of the FAD-binding pocket, as these features are critical for maintaining enzymatic function. Representative structural snapshots extracted from the later stages of the simulations (125-150 ns) are shown in Figure 3A, demonstrating the absence of large-scale unfolding or significant structural distortion among the different systems. These observations indicate that the catalytic microenvironment of GOx remains structurally intact despite variations in the surrounding simulation conditions. The residue of root mean square fluctuations (RMSF) profiles (Figure 3B) further reveal that the majority of GOx residues exhibit limited fluctuations, particularly in regions proximal to the FAD-binding pocket. The average RMSF values of the apo protein systems are summarized as follows: FAD@GOx (0.128 ± 0.003 nm), Ce + FAD@GOx (0.110 ± 0.002 nm), CS + FAD@GOx (0.116 ± 0.002 nm), PVA + FAD@GOx (0.105 ± 0.002 nm), and Ce + CS + PVA + FAD@GOx (0.118 ± 0.002 nm).

Importantly, key catalytic residues previously identified in the literature such as GLU412, HIS516, and HIS559, display relatively low RMSF values compared to surface-exposed and loop regions. This restrained flexibility suggests a stable catalytic microenvironment, which is essential for efficient electron and proton transfer during glucose oxidation 56. This observation is consistent with previous studies that describe these residues as critical components of the GOx catalytic machinery, in which HIS516/520 and HIS559/563 function as potential proton acceptors, while GLU412 stabilizes the hydrogen-bond network near the FAD moiety 57,58. Consistent with prior structural and mutagenesis studies, the reduced mobility of HIS516 and HIS559 observed in this work supports the notion that one of these histidine residues functions as the base catalyst in the overall catalytic cycle of GOx 27. Collectively, these results indicate that the essential dynamic features of the GOx active site are preserved, maintaining a catalytically competent configuration that is consistent with previously reported native GOx systems.

**Figure 4:**
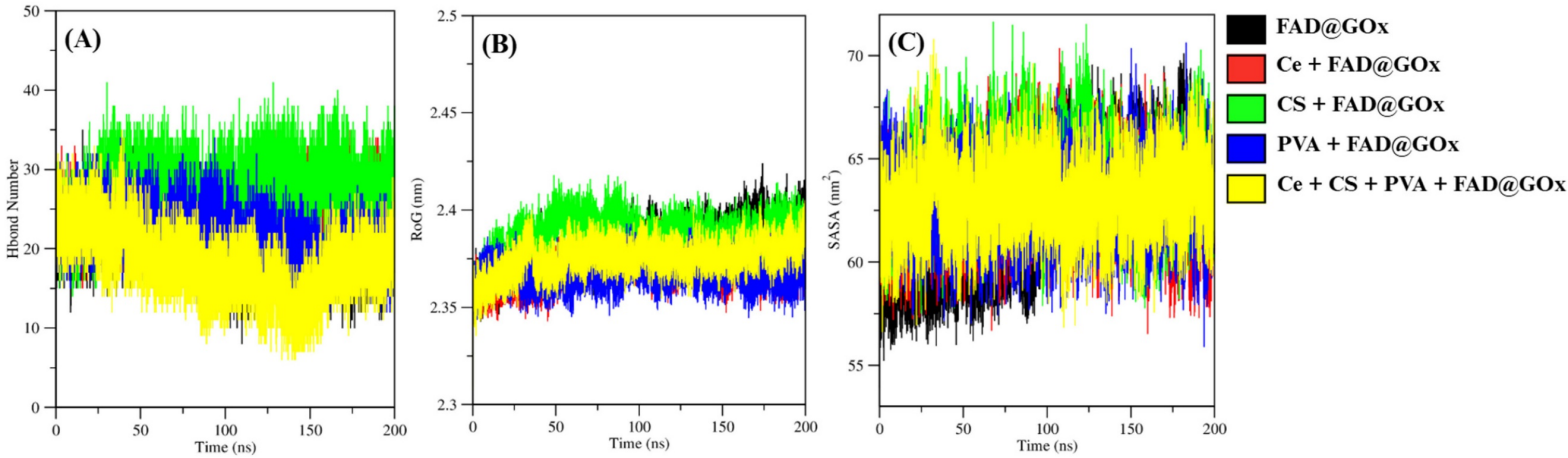
Various molecular dynamics-derived parameters of FAD@GOx systems: **(A)** Number of hydrogen bonds focusing on interactions between FAD and GOx, **(B)** RoG analyses for apo protein, and **(C)** SASA analysis active site for apo protein.

To further characterize the structural stability and intermolecular interactions of the FAD@GOx systems, hydrogen bond analysis, radius of gyration (RoG), and solvent-accessible surface area (SASA) were evaluated over the 0-200 ns simulation period (Figure 4). The hydrogen bond analysis was performed to evaluate the stability of interactions between the ligand-receptor environment 59,60. As shown in Figure 4A, the average number of hydrogen bonds involving the FAD cofactor remains relatively stable throughout the simulation period. The average number of hydrogen bonds involving the FAD cofactor in GOx was calculated over the 0-200 ns simulation period and summarized as follows: FAD@GOx (20.971 ± 0.026), Ce + FAD@GOx (23.429 ± 0.021), CS + FAD@GOx (27.155 ± 0.026), PVA + FAD@GOx (21.104 ± 0.023), and Ce + CS + PVA + FAD@GOx (18.059 ± 0.029). To further assess the persistence of these interactions, the hydrogen bond occupation percentages were calculated over the entire simulation timeframe. The results show high occupancy values for most systems, with FAD@GOx exhibiting 91.03% occupancy, followed by Ce + FAD@GOx (86.206%), CS + FAD@GOx (98%), PVA + FAD@GOx (38.583%), and Ce + CS + PVA + FAD@GOx (74.431%).

The radius of gyration (RoG) was used to evaluate the global compactness of the system during the simulations and surface accessible surface area (SASA) analysis reflect alterations in conformational stability and folding behavior throught the simulation time 61,62. As shown in Figure 4B and Figure 4C, all systems exhibit stable RoG values and SASA throughout the 0-200 ns simulation period, indicating the absence of significant structural expansion or collapse. The average RoG values of the apoprotein in detailed: FAD@GOx (2.381 ± 0.000 nm), Ce + FAD@GOx (2.372 ± 0.000 nm), CS + FAD@GOx (2.388 ± 0.000 nm), PVA + FAD@GOx (2.367 ± 0.000 nm), Ce + CS + PVA + FAD@GOx (2.375 ± 0.000 nm), and the average SASA values of he apoprotein in detailed : FAD@GOx (60.230 ± 0.022 nm), Ce + FAD@GOx (62.994 ± 0.013 nm), CS + FAD@GOx (64.402 ± 0.013 nm), PVA + FAD@GOx (62.887 ± 0.012 nm), Ce + CS + PVA + FAD@GOx (63.203 ± 0.012 nm).

**Figure 5:**
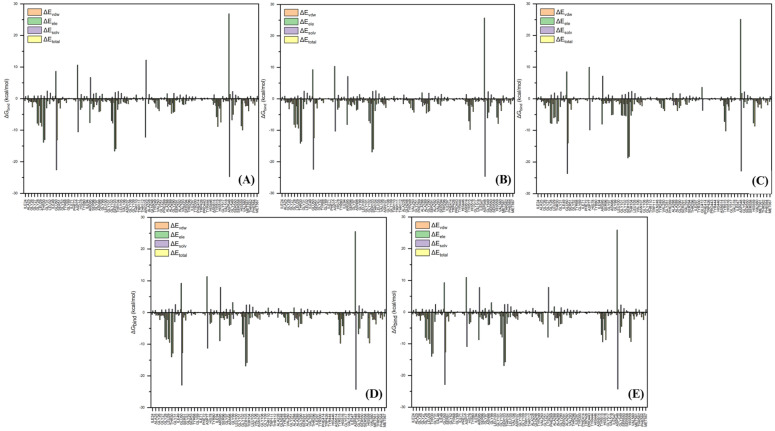
Peresidue energy decomposition profiles focus on interactions between FAD and GOx over the 125-150 ns; **(A)** FAD@GOx, **(B)** Ce + FAD@GOx, **(C)** CS + FAD@GOx, **(D)** PVA + FAD@GOx, and **(E)** Ce + CS + PVA + FAD@GOx.

Per-residue energy decomposition was employed as a complementary analysis to support the interpretation of the overall binding free energy (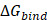), which serves as a key indicator of the interaction strength between FAD and GOx (Figure 5). In general, 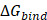
plays a crucial role in describing the thermodynamic favorability of ligand-receptor interactions, where more negative values correspond to spontaneous and more stonger binding process 63-65. As shown in Table 2, the binding affinity follows the order FAD@GOx < PVA + FAD@GOx < Ce + CS + PVA + FAD@GOx < Ce + FAD@GOx < CS + FAD@GOx. The analysis further highlights specific amino acid residues that consistently contribute significantly to the binding stabilization, with individual energy contributions of ≤ -1.00 kcal·mol^-1^ 66, underscoring their dominant role in maintaining FAD anchoring within the GOx active site. Detailed contributions of the individual energy components are summarized in Table S3. It should be noted that, in the present molecular dynamics simulations, cerium was represented as a single Ce atom to simplify the computational model. While this approach provides preliminary insight into the interaction behavior, it may not fully capture the structural and electronic characteristics of CeO_2_ NPs. Therefore, the use of a more representative CeO_2_ NPs model could offer a more realistic description of atomic-level interactions within the system.

### Ultraviolet visible spectrophotometry (UV-Vis) study

**Figure 6:**
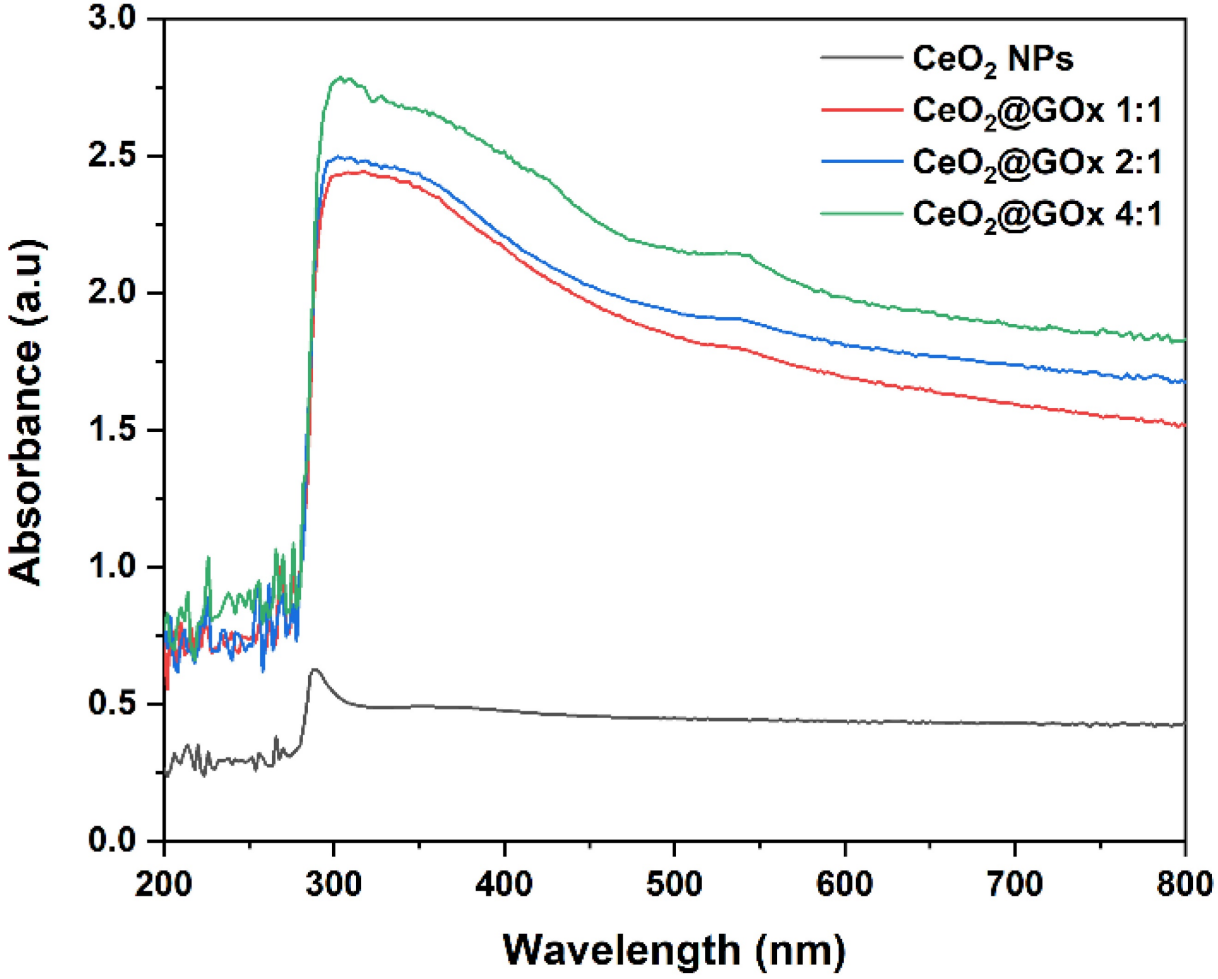
UV-Vis characterization spectrum of CeO_2_@GOx.

The denaturation of GOx upon adsorption onto nanostructured surfaces is generally associated with alterations in its molecular conformation, which may lead to partial or complete loss of enzymatic activity 67. Thus, UV-Vis spectroscopy was utilized to monitor potential alterations in the electronic and conformational structure of GOx during the modification process. UV-Vis was used to examine the possible conformation changes of GOx following the incorporation of CeO_2_ NPs. As shown in Figure 6, UV-Vis analysis shows a peak at a wavelength of 296 nm which indicates the presence of absorbance in CeO_2_ NPs 68,69. Furthermore, the presence of native GOx is indicated by an absorption band at ~380 nm, which is attributed to the polypeptide chains, while a slight red-shift toward ~530 nm can be associated with the flavin prosthetic group within the protein structure 70,67. Increasing the CeO_2_@GOx ratio from 1:1, 2:1, to 4:1 causes an increase in absorbance which indicates the concentration of CeO_2_ NPs 37,71.

### Fourier transform infrared spectroscopy (FT-IR) study

**Figure 7:**
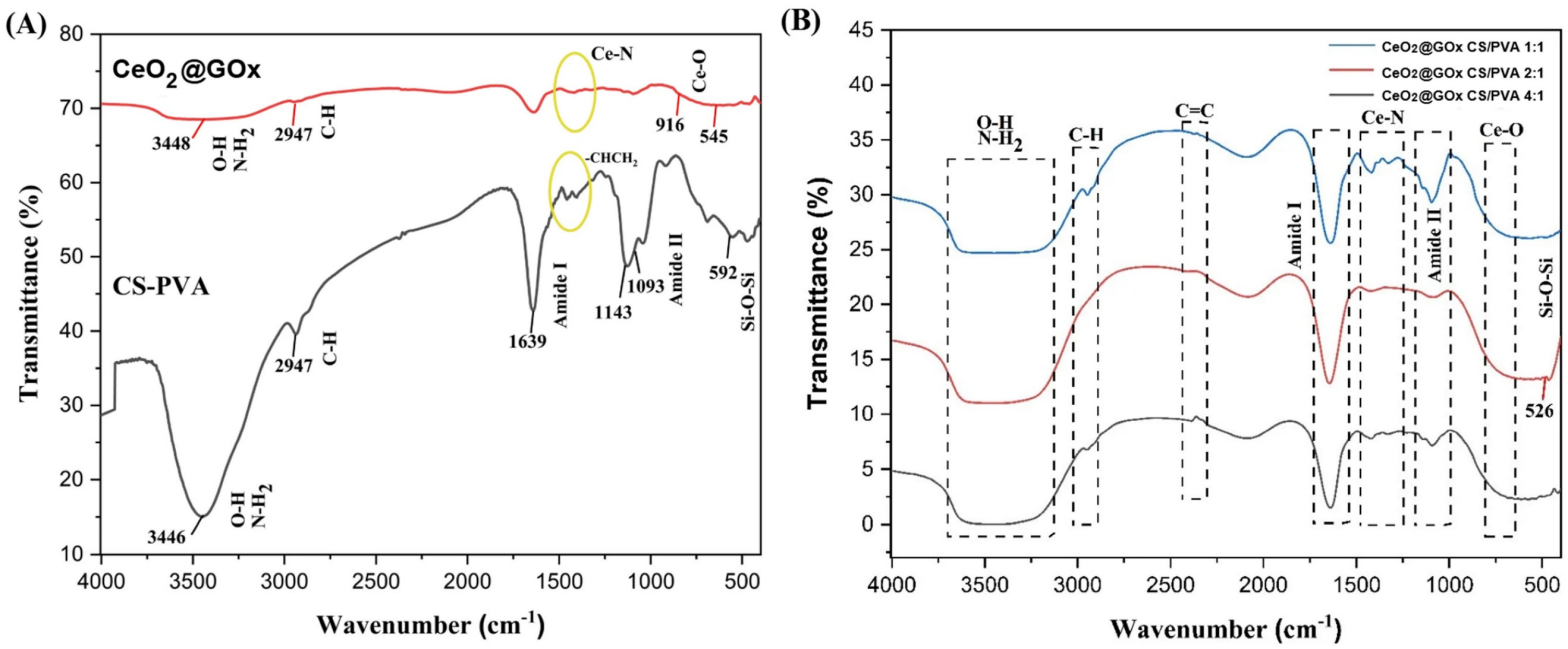
FT-IR characterization results; **(A)** Ce@GOx and CS/PVA; **(B)** Hydrogel variations of Ce@GOx CS/PVA 1:1, Ce@GOx CS/PVA 2:1, and Ce@GOx CS/PVA 4:1.

Fourier transform infrared (FT-IR) characterization was performed on CeO_2_@GOx CS/PVA hydrogels with mass ratios of 1:1, 2:1, and 4:1 in the range of 4000-500 cm^-1^. The FT-IR spectrum of CS/PVA exhibits broad absorption peaks at 3000-3700 cm^-1^, attributed to O-H and N-H_2_ stretching vibrations, along with C-H stretching vibrations at 2947 cm^-1^. The characteristic sharp peaks belonging to amide I and amide II bands, as well as bending vibrations corresponding to -CHCH_2_ (Figure 7A). The spectrum of CeO_2_@GOx shows a characteristic metal-oxygen stretching vibrations (Ce-O) at ~535 cm^-1^, while the band at 1035 cm^-1^ represented as O-Ce-O bond 72,73. As shown in Figure 7B, a weak band observed at 1000-1500 cm^-1^ occurs due to the complexation between Ce and amine groups in that area 74.

### Particle size analyzer (PSA) study

**Figure 8:**
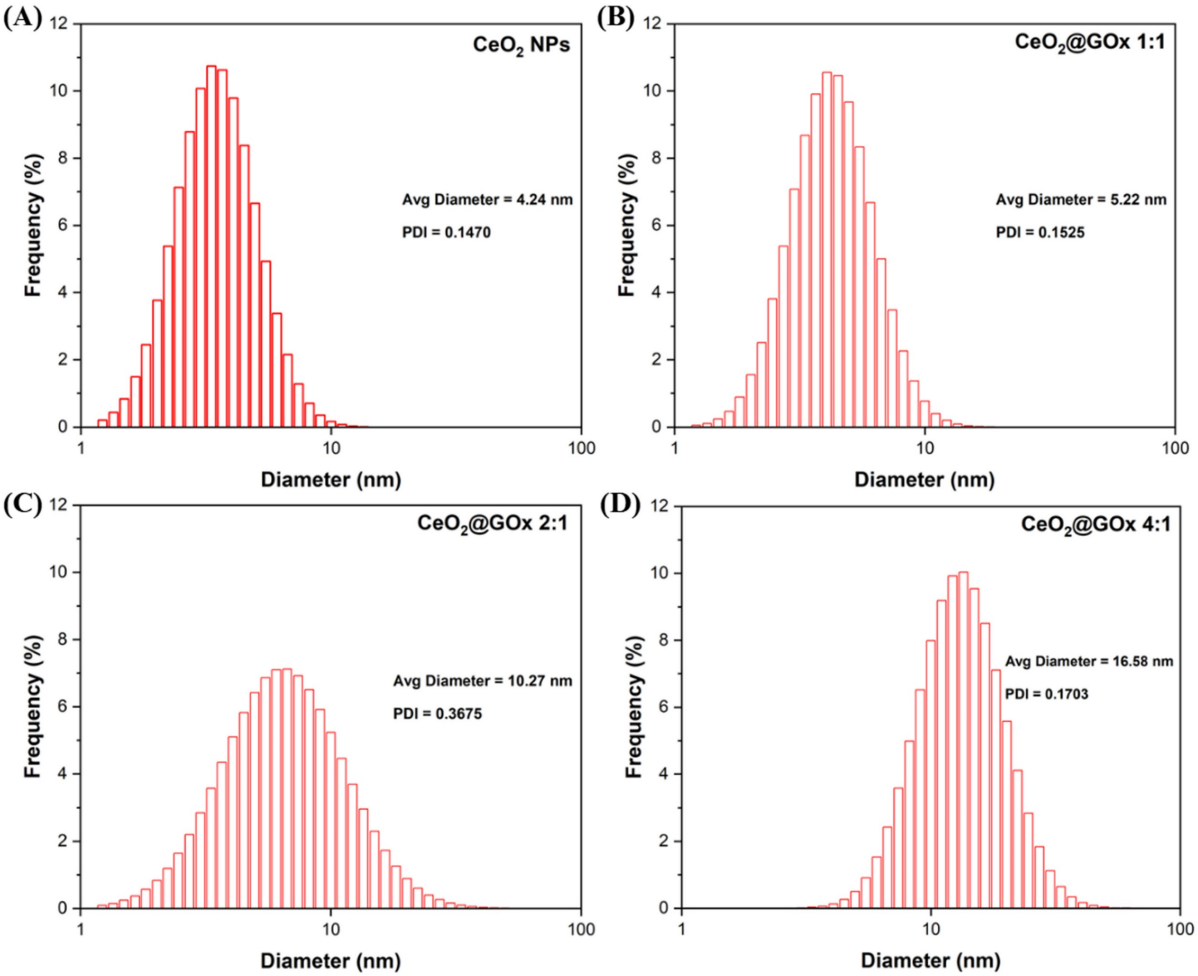
PSA characterization results; **(A)** CeO_2_ NPs, **(B)** CeO_2_@GOx 1:1, **(C)** CeO_2_@GOx 2:1, and **(D)** CeO_2_@GOx 4:1.

Characterization using Particle Size Analyzer (PSA) was performed to determine the range and distribution of particle size especially after the synthesis process of CeO_2_ NPs and functionalization of CeO_2_ NPs with GOx based on the principle of Brownian motion. According to the characterization with a measurement range of 1nm-1000nm, the average diameter of the CeO_2_ NPs sample (Figure 8A) was 7.76 nm with a Polydispersity Index (PDI) of 0.3668 indicating the successful formation of CeO_2_ NPs. The Ce@GOx 1:1 sample (Figure 8B) has an average diameter of 5.22 nm with a PDI of 0.1525. The CeO_2_@GOx 2:1 sample (Figure 8C) has an average diameter of 10.27nm with a PDI of 0.3675. The CeO_2_@GOx 4:1 sample (Figure 8D) has an average diameter of 16.58nm with a PDI of 0.1703. The CeO_2_@GOx 2:1 and CeO_2_@GOx 4:1 samples showed an average particle size that was in accordance with the recomended particle size range for transdermal route drug administration of 10nm-600nm 75. In addition, all four samples have a PDI value ≤ 0.3 which indicates a homogeneous particle size distribution and is acceptable for industrial drug applications 76.

### Degradation test

**Figure 9:**
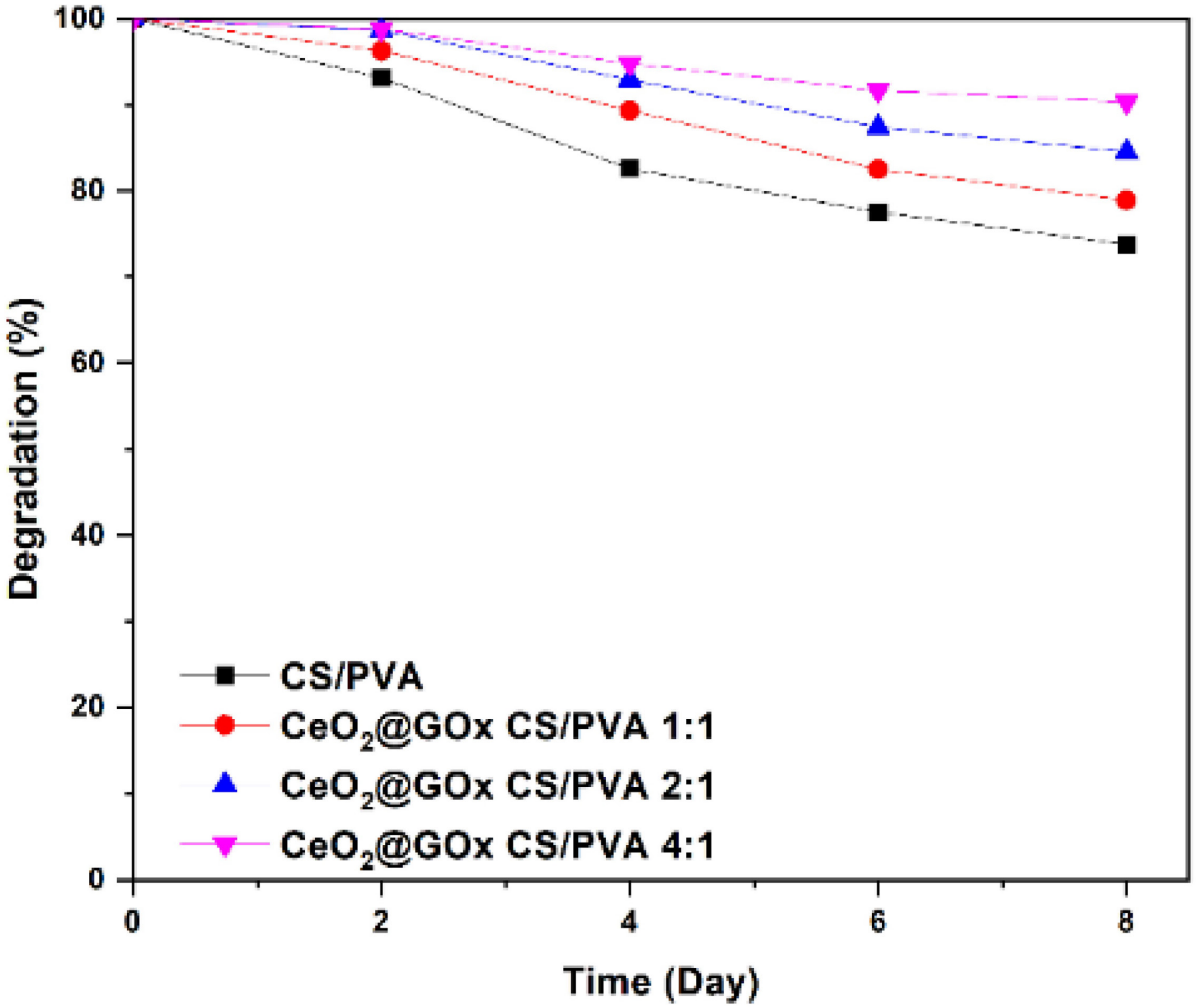
Degradation test of the CeO_2_@GOx hydrogel sample in CS/PVA for every variation.

The weight loss of the hydrogels was monitored in phosphate buffer saline (PBS) at room temperature over time. The hydrogel weight decreased significantly up to day 8, as shown in (Figure 9). CeO_2_@GOx CS/PVA 4:1 hydrogel resulted in degradation with the highest mass reduction of 90.3%. The degradation rate of CeO_2_@GOx CS/PVA 2:1 hydrogel was slightly lower, with approximately 84.575% of the initial weight remaining at day 8. The degradation rate of CeO_2_@GOx CS/PVA 1:1 hydrogel remained at 78.9%. Meanwhile, the CS/PVA hydrogel showed the lowest mass reduction, which was 73.7%. This mass reduction is due to the breakdown of the crosslinking bonds that hold the hydrogel structure 13. Degradation performance is very important in applying materials as wound healing in wounds, especially in diabetic wounds 77. The application of hydrogels in wound healing must have high physical resistance with low degradation rates to maintain their structure and function in cell and tissue regeneration for a certain period of time.

### Enzymatic activity (peroxidase and catalase)

**Figure 10:**
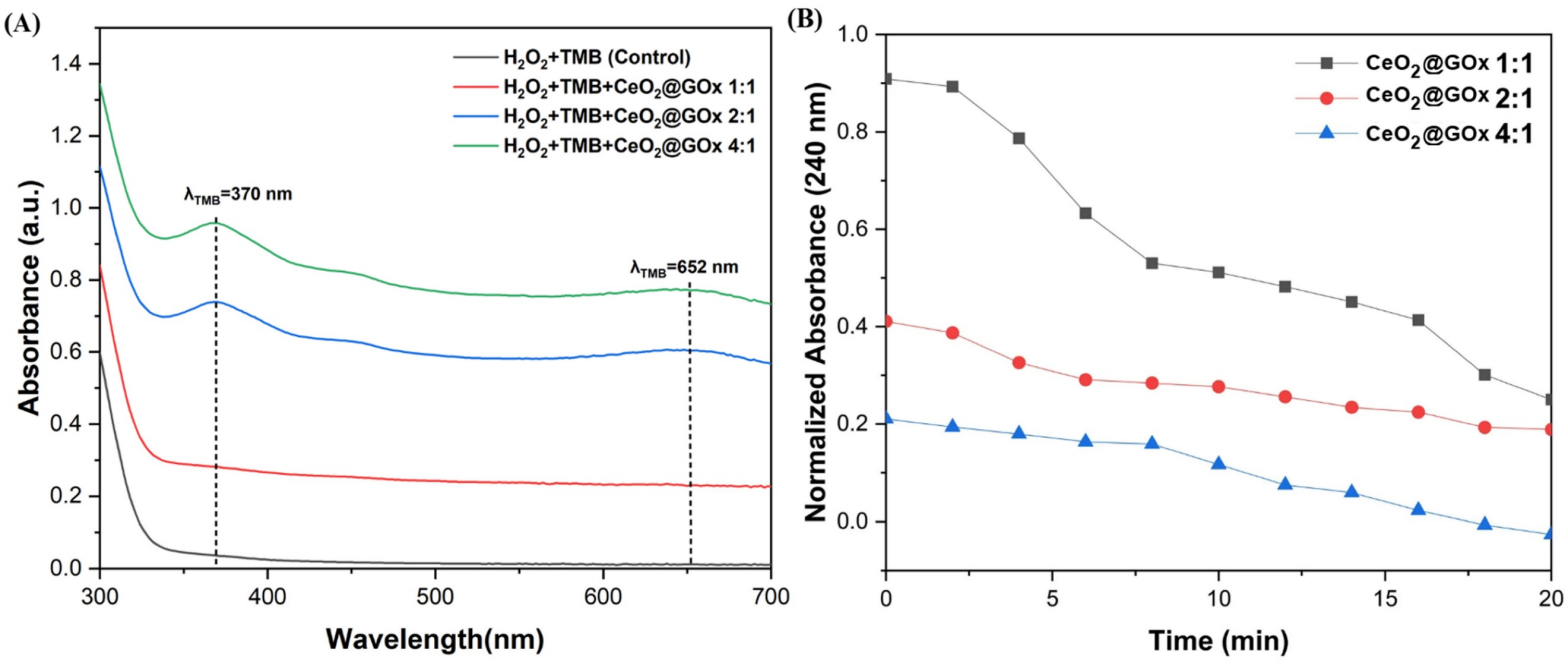
Enzymatic activity analysis; **(A)** Peroxidase, and **(B)** Catalase.

The enzymatic peroxidase activity of CeO_2_@GOx sample was tested through the oxidation rate of 3,3′,5,5′-tetramethylbenzidine (TMB). As shown in Figure 10A, the sample exhibited good affinity towards TMB in the presence of H2O_2_, as evidenced by the appearance of dual characteristic peaks at 370 nm and 652 nm, which are indicative of typical nanozyme behavior 78,79. Furthermore, the catalase-like activity of CeO_2_@GOx was demonstrated by the concentration-dependent degradation of H_2_O_2_, monitored via the decrease in absorbance at 240 nm (Figure 10B) 80. According to previous studies, CeO_2_ is capable of catalytically decomposing H_2_O_2_ to regenerate oxygen, thereby protecting GOx from oxidative denaturation 81. In the CeO₂@GOx system, glucose oxidation proceeds through the canonical catalytic cycle of glucose oxidase (GOx). According to Ernst *et al.* 82, glucose molecules are oxidized by GOx in its flavin adenine dinucleotide (FAD) form, producing gluconolactone and reducing GOx(FAD) to GOx(FADH₂). The gluconolactone intermediate subsequently hydrolyzes to form gluconic acid. The reduced enzyme is then re-oxidized by molecular oxygen, leading to the generation of hydrogen peroxide (H₂O₂) as a key reaction intermediate. In the presence of CeO₂, the locally generated H₂O₂ can be dynamically regulated through its peroxidase- and catalase-like activities, thereby mitigating oxidative stress and preserving the structural integrity of GOx. Here, the catalytic cycle of GOx is based on previous research 82,83:

GOx(FAD) + β-D-glucose → GOx(FADH₂) + gluconolactone (11)

GOx(FADH₂) + O_2_ → GOx(FAD) + H_2_O_2_ (12)

H_2_O_2_ → O_2_ + 2e^-^ + 2H^+^ (13)

### Antimicrobial assay

Disc diffusion assay (DDA) results for CeO_2_@GOx sample variations and an antimicrobial agent named gentamicin against the growth of Gram-negative bacteria *Escherichia coli* ATCC 25922 and Gram-negative bacteria *Staphylococcus aureus* ATCC 25923 are presented in Table 3 and Table 4. The results indicate moderate antimicrobial activity from CeO_2_@GOx sample variations with all average inhibition zones not lower than 6 mm 84,85. Yet the results are still far from a commercial antibacterial agent such as gentamicin, which shows a strong antimicrobial activity. In addition, CeO_2_ NPs can bind to microbial proteins and disrupt essential functions. The released cerium ions interfere with respiration and electron flow in microbial cells. CeO_2_ NPs are also known to interact with thiol groups (-SH) and membrane transporters or porins, hindering the transport of nutrients into the cell. Another critical antimicrobial mechanism is oxidative stress 86,87. The reversible cycling between Ce(III) and Ce(IV) states on the surface of microbial membranes leads to the generation of reactive oxygen species (ROS). These ROS attack vital cellular components such as lipids, proteins, polysaccharides, and nucleic acids, resulting in functional damage and eventual microbial cell death. Although the CeO_2_@GOx CS/PVA nanocomposite demonstrated antimicrobial activity, the observed effects were categorized as moderate. This outcome suggests several possible limitations that warrant further evaluation. First, the enzymatic activity of glucose oxidase (GOx) might have been partially compromised during nanoparticle functionalization or matrix incorporation, potentially reducing hydrogen peroxide (H₂O₂) generation. The limited production of H₂O₂ would weaken the oxidative stress required for effective microbial inhibition. Additionally, the redox capacity of cerium oxide nanoparticles depends heavily on the Ce(III)/Ce(IV) ratio, which may not have been optimized, thus diminishing reactive oxygen species (ROS) generation 88,89.

Another contributing factor could be the matrix composition. While chitosan and PVA provide structural and biocompatible support, their interaction with CeO_2_ NPs and GOx may restrict the diffusion or release of active agents. A high ratio of PVA, for instance, could create a dense polymer network that hinders effective contact with microbial cells. Furthermore, the release profile of H₂O₂ or Ce ions may be too slow to induce significant antimicrobial effects within the test duration. Environmental factors during testing, such as the pH or availability of glucose, may also have played a role. GOx activity is substrate-dependent; thus, insufficient glucose concentration in the medium would limit the enzymatic reaction and subsequent H₂O₂ production. The type and resistance level of the tested microbial strains could further influence the outcome, especially if the bacteria possess tolerance mechanisms against oxidative stress. To improve antimicrobial performance, future work should consider optimizing the GOx and CeO_2_ NPs loading ratios, adjusting the CS/PVA composition, and confirming enzymatic and redox activities through H₂O₂ and ROS quantification assays. Additionally, pre-incubation strategies or combining this system with other antimicrobial agents may offer synergistic effects to enhance overall efficacy.

### *In vivo* test results

**Figure 13:**
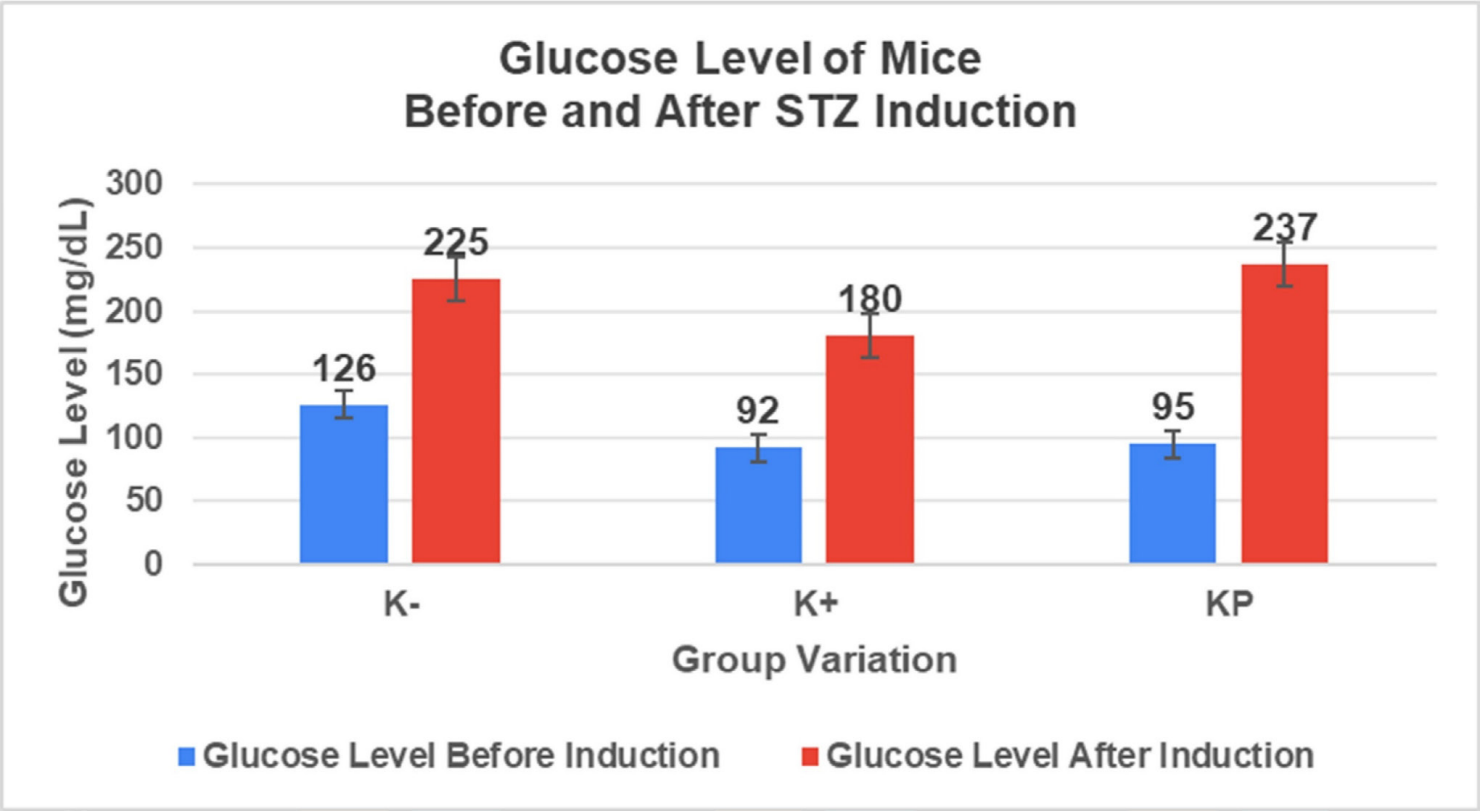
Diabetes induction in mice.

The results showed a significant increase in blood glucose levels in various treatment groups after STZ induction. Mice are declared diabetic if blood glucose levels exceed 175 mg/dL 90,91. Based on these criteria and Figure 13, it can be concluded that the administration of STZ is effective in inducing diabetic conditions in mice, with blood glucose levels in all groups showing hyperglycemia for 5 days. Visual observation results in (Table 5) show KP as the group with wound healing rate close to KN, indicating the use of CeO_2_@GOx CS/PVA 4:1 hydrogel as a sample with superior healing effect compared to the use of conventional drugs. Nevertheless, comprehensive histopathological analysis is required to further validate the quality of tissue regeneration, modulation of the inflammatory response, and collagen remodeling at the microscopic level. Such investigations will provide deeper insight into the therapeutic mechanism and further strengthen the clinical potential of the developed hydrogel system.

## Conclusion

CeO_2_@GOx hydrogels in CS/PVA solution were successfully synthesized through four main stages, namely CeO_2_ NPs synthesis, CS/PVA hydrogel formation, CeO_2_@GOx incorporation, and composite hydrogel formation by freeze-thawing method. Characterization analysis showed that increasing the CeO_2_@GOx ratio from 1:1, 2:1, to 4:1 led to an increase in absorbance, indicating an increasing Ce concentration. Degradation and enzymatic activity tests showed that CeO_2_@GOx CS/PVA hydrogels have good potential as wound healing materials. *In vivo* tests on mice with diabetic wound models showed that the use of CeO_2_@GOx 4:1 CS/PVA hydrogel provided a better healing effect compared to conventional drugs, with wounds completely closed on day 7. The results of this study indicate that CeO_2_@GOx CS/PVA hydrogel has potential as an innovation in diabetic wound management.

## Supplementary Material

Supplementary figures and tables.

## Figures and Tables

**Figure 11:**
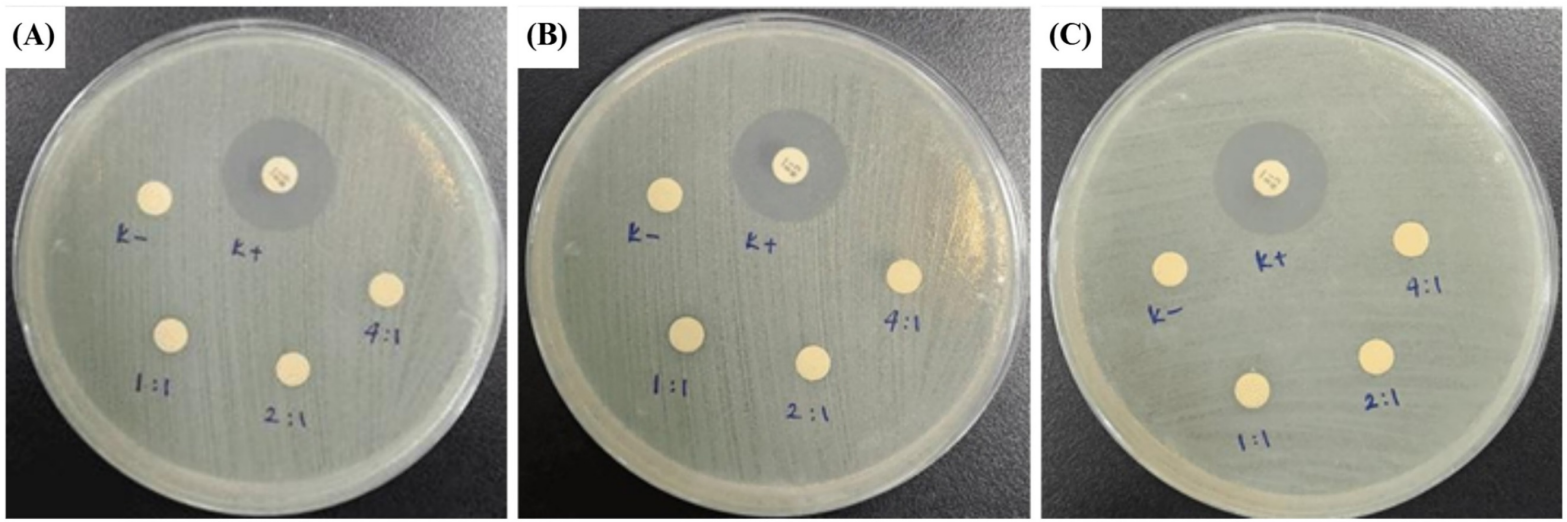
Zone of inhibition test results against the growth of *Escherichia coli* ATCC 25922; **(A)** First-replication, **(B)** Second-replication, and **(C)** Third-replication.

**Figure 12:**
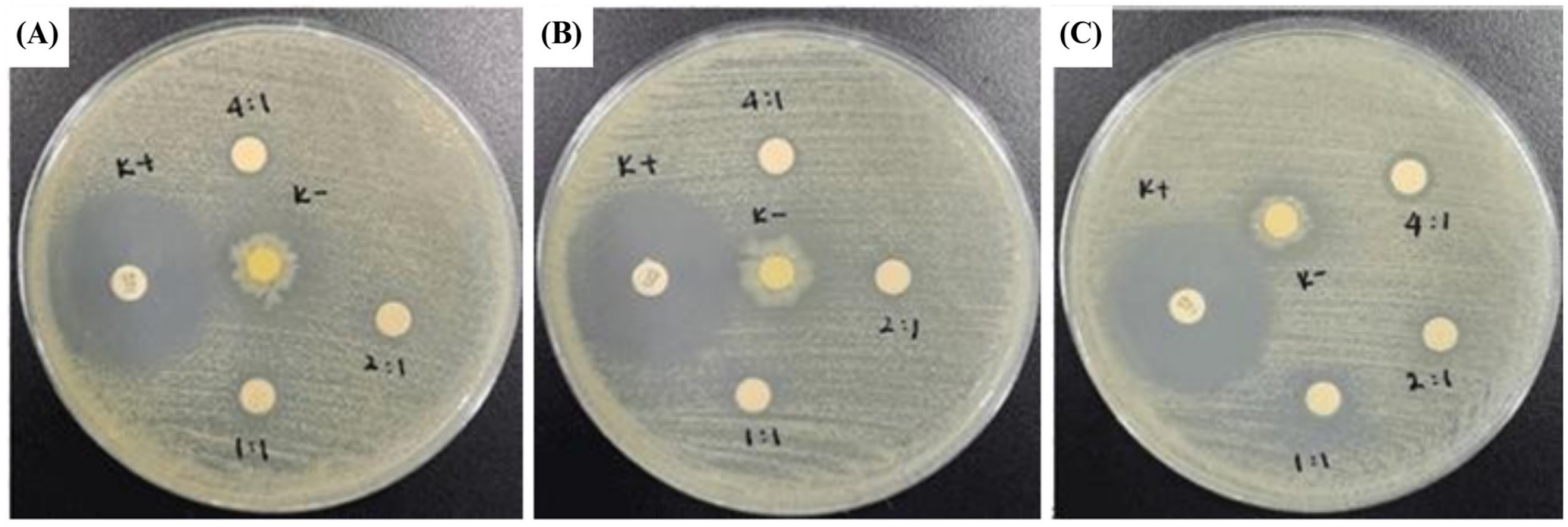
Zone of inhibition test results against the growth of *Staphylococcus aureus* ATCC 25923; **(A)** First-replication, **(B)** Second-replication, and **(C)** Third-replication.

**Table 1:** Global reactivity descriptors of Ce₄O₈, CS, and PVA

Descriptors	Ce_4_O_8_	CS	PVA
Electronegativity (χ) (eV)	0.171	0.114	0.108
Chemical potential (μ) (eV)	-0.171	-0.114	-0.108
Chemical Hardness (η) (eV)	0.098	0.129	0.139
Softness (S) (eV^-1^)	5.102	3.876	3.597
Electrophilicity index (ω) (eV)	0.149	0.050	0.042
ΔNmax	1.745	0.884	0.777

**Table 2:** Energy components (kcal mol^-1^) calculated using the MM-GBSA approach over the 125-150 ns. Values are reported as mean ± standard error of the mean (SEM), with a specific focus on interactions between FAD and GOx

Component	ΔE*_vdw_*	ΔE*_ele_*	ΔG*_gas_*	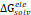	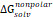	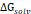	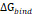
FAD@GOx	-88.32 ± 0.13	78.61 ± 0.41	-9.72 ± 0.47	-67.32 ± 0.17	-11.72 ± 0.00	-79.04 ± 0.17	-88.76 ± 0.50
Ce + FAD@GOx	-87.31 ± 0.13	81.95 ± 0.55	-5.36 ± 0.60	-64.06 ± 0.02	-11.37 ± 0.00	-75.43 ± 0.02	-80.79 ± 0.60
CS + FAD@GOx	-82.14 ± 0.02	90.89 ± 0.27	8.75 ± 0.34	-69.83 ± 0.46	-10.95 ± 0.00	-80.78 ± 0.46	-72.03 ± 0.57
PVA + FAD@GOx	-86.37 ± 0.14	83.19 ± 0.65	-3.19 ± 0.70	-70.37 ± 0.30	-11.53 ± 0.00	-81.90 ± 0.30	-85.08 ± 0.76
Ce + CS + PVA + FAD@GOx	-83.88 ± 0.15	73.01 ± 0.51	-10.87 ± 0.55	-61.09 ± 0.20	-11.16 ± 0.00	-72.24 ± 0.20	-83.11 ± 0.59

**Table 3:** Inhibition zone diameter against the growth of *Escherichia coli* ATCC 25922

No.	Sample Code	Inhibition Zone Diameter (mm)			Average Inhibition Zone (mm)
		D1	D2	D3	
1	K(+)*	20.35	19.86	19.90	20.10
2	K(-)*	0	0	0	0
3	CeO_2_@GOx 1:1	7.36	7.07	7.63	7.35
4	CeO_2_@GOx 2:1	6.90	6.81	6.71	6.81
5	CeO_2_@GOx 4:1	6.45	6.44	6.71	6.54

*K(+) : Gentamicin K(-) : Aquades

**Table 4:** Inhibition zone diameter against the growth of *Staphylococcus aureus* ATCC 25923

No.	Sample Code	Inhibition Zone Diameter (mm)			Average Inhibition Zone (mm)
		D1	D2	D3	
1	K(+)*	30.65	29.73	24.55	30.19
2	K(-)*	0	0	0	0
3	CeO_2_@GOx 1:1	7.97	8.63	8.38	8.33
4	CeO_2_@GOx 2:1	7.59	7.88	7.72	7.73
5	CeO_2_@GOx 4:1	7.85	8.02	8.97	8.28

*K(+) : Gentamicin K(-) : Aquades

**Table 5:** Results of visual observation of diabetic wound healing in mice

Treatment group	Day 1	Day 2	Day 3	Description
KN	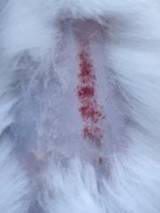	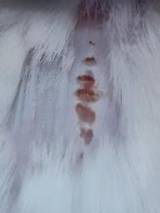	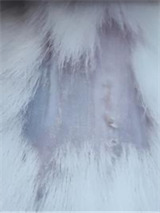	Non-diabetic, wounded, and untreated. Mice showed no inflammation and proliferation was rapid until the wound was completely closed by day 7.
K-	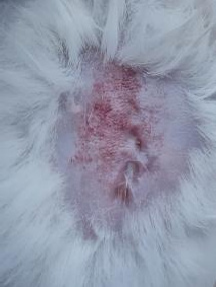	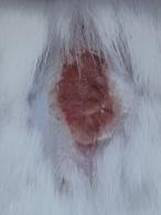	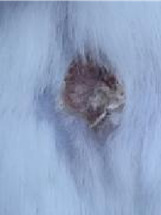	Diabetic, wounded, and untreated. The mice showed prolonged inflammation and proliferation until the wound had not entered the maturation stage by day 7.
K+	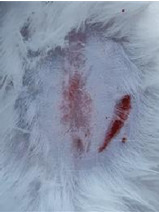	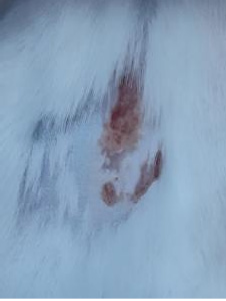	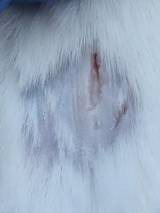	Diabetics, wounded, and administered 10% betadine ointment (Transdermal). The mice showed no inflammation and moderate proliferation until the 7^th^ day when the wound entered the maturation stage.
KP	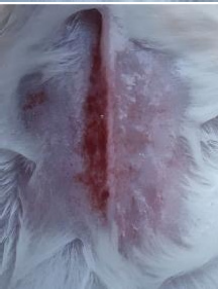	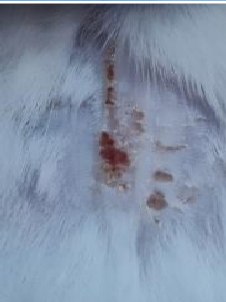	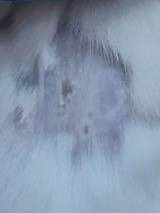	Diabetics, wounded, and administered CeO_2_@GOx CS/PVA composite hydrogel variation 4:1. The mice showed no inflammation and proliferation was rapid until the wound was completely closed on the 7^th^ day.
