# Supplementary material for: A Glucose-Responsive CeO₂@GOx Nanozyme Embedded in Chitosan/PVA Hydrogel for Accelerated Diabetic Wound Healing: from Molecular Simulations to In Vivo Validation
Source: Nanotheranostics. 2026 Mar 17;10:75–92. doi: 10.7150/ntno.132646 (PMC13143520; doi:10.7150/ntno.132646)
Supplement: Supplementary file 1 — Supplementary figures and tables. [file ntnov10p0075s1.pdf]

Supplementary Materials

A Glucose-Responsive CeO_2@GOx Nanozyme Embedded in Chitosan/PVA Hydrogel for Accelerated Diabetic Wound Healing: From Molecular Simulations to In Vivo Validation

Lidia Grace Naomi[1], Dino Pati Putra[1], Rangga Adhi Prastika[1], Azizah Mirza Kautsari[1], Dewi Sintawati Try Sutrisno[1], Suhailah Hayaza[1,2*], Melisa Intan Barliana[3,5], Windri Handayani[4] , Nik Ahmad Nizam Nik Malek[6] , Inna Syafarina[7]

[1]Nanotechnology Engineering, Faculty of Advanced Technology and Multidiscipline, Universitas Airlangga, Surabaya 60115, Indonesia
[2]Airlangga Functional Nanomaterials Research Group, Faculty of Advanced Technology and Multidiscipline, Universitas Airlangga, Surabaya 60115, Indonesia
[3]Department of Biological Pharmacy, Faculty of Pharmacy, Universitas Padjadjaran, Sumedang, Indonesia
[4]Department of Biology, Faculty of Mathematics and Natural Sciences, Universitas Indonesia, Indonesia
[5]Center of Excellence for Pharmaceutical Care Innovation, Faculty of Pharmacy, Universitas Padjadjaran, Sumedang, Indonesia
[6]Center for Sustainable Nanomaterials (CSNano), Ibnu Sina Institute for Scientific and Industrial Research (ISI-ISIR), Universiti Teknologi Malaysia, 81310 Johor, Malaysia
[7]Research Center for Computing, National Research and Innovation Agency, Jl. Raya Jakarta Bogor KM 46, Cibinong, Indonesia.

*Corresponding Author: suhailah@ftmm.unair.ac.id

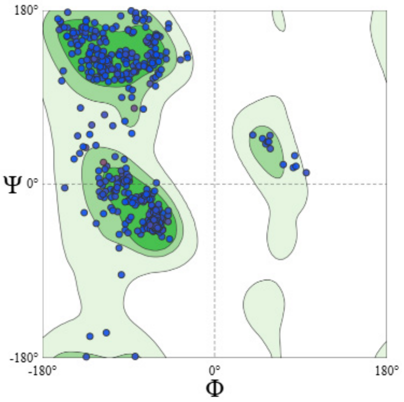

Figure S1. Ramachandran plot of the protein structure, showing the distribution of backbone dihedral angles (Φ and Ψ). The green shaded regions represent the most favored and additionally allowed conformational regions, while the blue dots correspond to the dihedral angles of individual amino acid residues. The majority of residues are located within the favored regions, indicating good stereochemical quality of the protein structure.

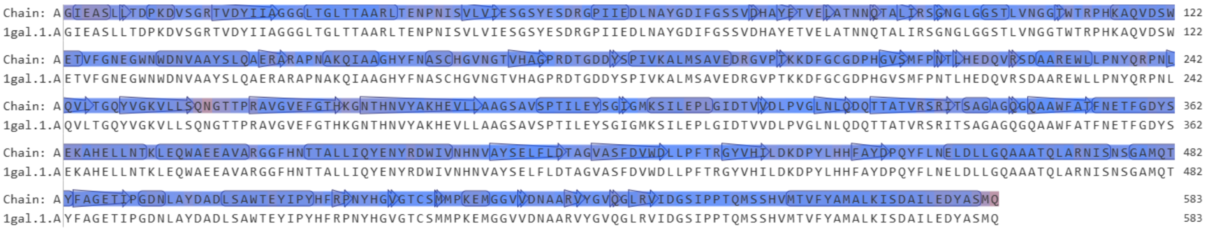

Figure S2. Amino acid sequence representation of GOx (Chain A) highlighting the coverage and conserved regions used in the simulation. The highlighted segments indicate residues involved in the structural model and interactions with FAD, ensuring the structural integrity of the active site.

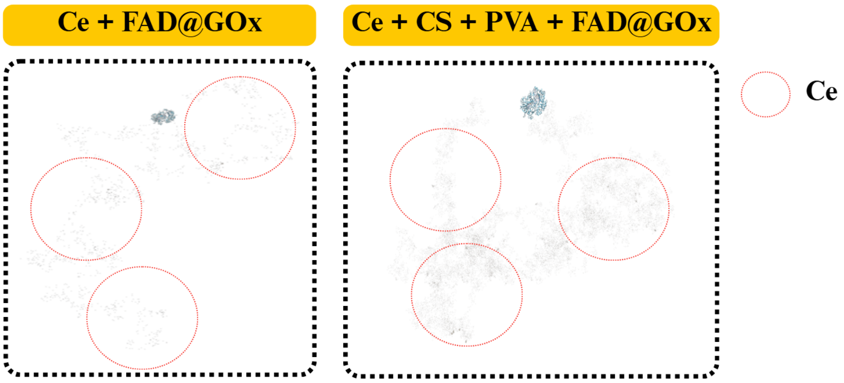

Figure S3. Comparison of the spatial distribution of cerium (Ce) in the Ce + FAD@GOx and Ce + CS + PVA + FAD@GOx systems. The highlighted regions indicate Ce-rich areas, showing that the incorporation of CS and PVA influences the dispersion and localization of Ce around the FAD@GOx system.

Table S1. Binding pocket characteristics predicted by PrankWeb, showing the top-ranked binding site features used for docking site selection.

Pocket Rank	Glucose oxidase		
	1	2	3
Pocket score	71.40	1.44	1.00
Probability score	0.990	0.019	0.007
Amino acid count	57	9	8

Amino acid residues	ALA25, GLY26, GLY28, LEU29, THR30, ILE49, GLU50, SER51, TYR68, PHE72, HIS78, TYR80, ILE94, ARG95, SER96, GLY97, GLY102, SER103, THR104, VAL106, ASN107, GLY108, GLY109, THR110, TYR249, VAL250, ALA288, ALA289, GLY290, SER291, ALA292, VAL293, THR296, ILE297, TYR300, THR331, GLN347, PHE414, ASP416, SER422, ASP424, TRP426, PRO443, HIS446, PHE448, TYR450, ARG512, PRO513, ASN514, TYR515, HIS516, GLY517, GLY549, HIS559, VAL560, MET561, PHE564	MET305, ILE308, LEU324, ARG383, GLY384, PHE386, HIS387, TYR435, HIS437	LYS116, TRP133, ALA137, LEU141, SER163, CYS164, GLY205, CYS206

Table S2. All average component of the MD analysis at 0-200 ns.

MD Analysis	FAD@GOx	Ce + FAD@GOx	CS + FAD@GOx	PVA + FAD@GOx	Ce + CS + PVA + FAD@GOx
RMSD (all atoms)	0.226 ± 0.000	0.298 ± 0.000	0.222 ± 0.000	0.223 ± 0.000	0.328 ± 0.000
RMSD (backbone)	0.172 ± 0.000	0.147 ± 0.000	0.169 ± 0.000	0.138 ± 0.000	0.155 ± 0.000
RMSD (Cofactor)	0.077 ± 0.000	0.569 ± 0.000	0.129 ± 0.000	0.060 ± 0.000	0.100 ± 0.000
RMSF (apo protein)	0.128 ± 0.003	0.110 ± 0.002	0.116 ± 0.002	0.105 ± 0.002	0.118 ± 0.002
RoG (apo protein)	2.381 ± 0.000	2.372 ± 0.000	2.388 ± 0.000	2.367 ± 0.000	2.375 ± 0.000
SASA (active site apo protein)	60.230 ± 0.022	62.994 ± 0.013	64.402 ± 0.013	62.887 ± 0.012	63.203 ± 0.012
Hbond (cofactor-protein)	20.971 ± 0.026	23.429 ± 0.021	27.155 ± 0.026	21.104 ± 0.023	18.059 ± 0.029
% Hbond occupation (cofactor-protein)	91.03%	86.206%	98%	38.583%	74.431%

Table S3. Detail of peresidue energy component for the MM-GBSA calculations at 125-150 ns.

FAD@GOx				
Residue	ΔE_{vdw}	ΔE_{ele}	ΔE_{sol}	ΔE_{total}
ILE24	-0.07242	-0.69969	0.69751	-0.07461
ALA25	-0.43151	0.94582	-1.18751	-0.6732
GLY26	-0.97007	-0.70309	-1.01864	-2.6918
GLY27	-1.08113	0.87842	-0.89074	-1.09344
GLY28	-1.49095	-7.82223	0.8802	-8.43297
LEU29	-2.25288	-7.5746	1.05818	-8.7693
THR30	0.03538	-13.85558	0.79635	-13.02385
GLY31	-0.18811	-2.9892	2.43762	-0.73968
LEU32	-0.10875	-1.67626	1.79148	0.00647
ILE49	-0.42895	0.69039	-0.84508	-0.58364
GLU50	0.83509	8.69145	-22.62593	-13.09939
SER51	-1.45461	-1.25923	-0.34896	-3.0628
GLY52	-0.23614	-0.29817	0.50818	-0.02612
TYR68	-0.65278	-0.27905	-0.39872	-1.33054
GLY69	-0.02716	-0.15601	0.13496	-0.04822
ILE71	-0.04061	0.0505	-0.0539	-0.04401
PHE72	-0.43061	-0.11526	-0.2915	-0.83737
ASP77	-0.06861	10.63383	-10.55009	0.01512
HIS78	-0.71457	-3.45764	1.33782	-2.83439
TYR80	-0.42877	-0.77371	0.74573	-0.45675
ILE94	-0.22937	0.71395	-0.70546	-0.22088
ARG95	-0.62986	-7.66049	6.69778	-1.59257
SER96	-0.79585	-3.3612	1.51497	-2.64208
GLY97	-0.89718	1.75098	-1.97996	-1.12616
ASN98	-0.52675	-4.1716	0.75855	-3.9398
GLY99	-0.60534	1.41035	-0.96385	-0.15884
LEU100	-0.30013	-0.79674	0.11907	-0.97779
GLY101	-0.42984	-0.33358	0.08446	-0.67896
GLY102	-1.23576	-7.04073	0.50414	-7.77234
SER103	-1.25453	-16.63323	2.00722	-15.88055
THR104	-0.55426	-3.46349	2.3031	-1.71466
LEU105	-0.1763	-1.52414	1.68991	-0.01052
VAL106	-0.85682	-1.08166	0.62895	-1.30953
ASN107	-1.53345	0.53717	-0.67483	-1.67111
GLY108	-0.2224	-0.26267	0.0957	-0.38938
GLY109	-0.10144	-0.13712	0.31425	0.07569
THR110	-0.55274	1.17925	-1.0292	-0.40269
TRP111	-0.06269	0.10111	-0.21313	-0.17471
THR112	-0.04309	0.4968	-0.58915	-0.13544
ARG225	-0.05465	-12.25851	12.22288	-0.09028
ALA228	-0.05754	-1.61409	1.35791	-0.31372
GLN248	-0.29227	0.38992	-0.46809	-0.37045
TYR249	-1.09447	-1.48119	-0.19351	-2.76917
VAL250	-0.51977	-3.17736	-0.19582	-3.89295

GLY251	-0.07698	0.28246	-0.29723	-0.09175
PHE269	-0.04455	-0.37352	0.3528	-0.06527
ALA288	-0.80326	1.60179	-2.44805	-1.64952
ALA289	-1.91508	-0.81685	-1.94948	-4.68141
GLY290	-1.40158	-4.38992	1.68383	-4.10767
SER291	-0.37992	-0.59586	0.61658	-0.3592
ALA292	-0.1099	-1.1359	1.22174	-0.02406
VAL293	-0.72378	-1.78133	0.54826	-1.95684
SER294	-0.13367	-1.72293	1.18294	-0.67367
THR296	-0.04274	-0.68534	0.60618	-0.12189
ILE297	-0.31819	-0.78904	0.63171	-0.47552
TYR300	-0.0305	-0.5713	0.56646	-0.03534
PHE414	-0.02183	-0.04211	-0.01096	-0.0749
TRP426	-0.16116	0.19935	-0.57254	-0.53435
PRO443	-0.02553	0.19091	-0.19844	-0.03306
TYR444	-0.05352	0.23899	-0.2315	-0.04603
HIS446	-0.12212	-0.01296	-0.06263	-0.1977
ASN514	-0.67023	-1.87312	1.2569	-1.28645
TYR515	-2.28912	-5.77184	-0.81819	-8.87915
HIS516	-2.34857	-3.09294	-2.04714	-7.48864
GLY517	-0.12587	0.74445	-0.9986	-0.38002
VAL518	-0.34579	-0.75587	0.33992	-0.76174
ASP548	-0.78761	26.86401	-24.73645	1.33995
GLY549	-0.48221	-6.8009	2.271	-5.0121
SER550	-0.12744	-2.08548	1.15907	-1.05384
SER558	-0.11793	0.68979	-0.42381	0.14806
HIS559	0.10779	-8.64817	-1.41943	-9.95982
VAL560	-0.54887	-0.40697	-1.41604	-2.37188
MET561	-2.24242	0.5268	-2.19825	-3.91386
THR562	-0.04756	0.76303	-0.80725	-0.09179
PHE564	-1.51369	0.2248	-0.83669	-2.12558
MET567	-0.0833	-0.93536	0.7752	-0.24346
Ce + FAD@GOx				
Residue	ΔE_{vdw}	ΔE_{ele}	ΔE_{sol}	ΔE_{total}
ILE24	-0.07859	-0.59804	0.58517	-0.09147
ALA25	-0.48644	0.88737	-1.25613	-0.85519
GLY26	-1.09075	-1.06487	-1.07029	-3.22591
GLY27	-1.10826	0.30001	-0.78379	-1.59204
GLY28	-1.527	-8.1446	0.59988	-9.07173
LEU29	-2.2877	-8.04712	0.97987	-9.35495
THR30	0.14603	-14.15856	0.49537	-13.51715
GLY31	-0.20894	-3.15882	2.47074	-0.89702
LEU32	-0.11217	-1.66347	1.85095	0.07531
ILE49	-0.4649	0.94478	-1.02119	-0.54131
GLU50	0.70733	9.30192	-22.39232	-12.38307
SER51	-1.46659	-1.33505	-0.17116	-2.9728
GLY52	-0.20785	-0.14206	0.35296	0.00304

TYR68	-0.59106	-0.44164	-0.2213	-1.254
GLY69	-0.02534	0.06588	-0.08175	-0.04121
ILE71	-0.05629	0.15518	-0.15744	-0.05855
PHE72	-0.44942	-0.10364	-0.42205	-0.97511
ASP77	-0.08916	10.35602	-10.29923	-0.03238
HIS78	-0.55178	-3.3662	1.19059	-2.72739
TYR80	-0.44457	-0.53454	0.55055	-0.42856
ILE94	-0.13482	0.63257	-0.55974	-0.06199
ARG95	-0.65898	-8.18619	7.11006	-1.73511
SER96	-0.73153	-1.95053	0.41561	-2.26645
GLY97	-1.02851	0.68229	-1.49566	-1.84188
ASN98	-0.47725	-3.62021	0.75924	-3.33822
GLY99	-0.45171	1.43167	-0.62185	0.35811
LEU100	-0.25038	-1.17063	0.46775	-0.95325
GLY101	-0.36669	-0.12438	-0.07014	-0.56122
GLY102	-1.21286	-6.99297	0.48444	-7.7214
SER103	-1.48329	-16.91523	2.38926	-16.00926
THR104	-0.53092	-3.77796	2.61448	-1.6944
LEU105	-0.16885	-1.41817	1.65542	0.06839
VAL106	-0.88402	-1.03872	0.44585	-1.47689
ASN107	-1.98644	0.70324	-1.49806	-2.78125
GLY108	-0.26759	-0.22365	2.28701E-4	-0.49101
GLY109	-0.10461	-0.08054	0.24277	0.05761
THR110	-0.36243	0.83989	-0.8219	-0.34444
TRP111	-0.046	0.24873	-0.33429	-0.13155
THR112	-0.03171	0.17254	-0.22161	-0.08078
ALA228	-0.06504	-1.59882	1.27582	-0.38804
GLN248	-0.2898	0.42599	-0.53229	-0.39611
TYR249	-1.1539	-1.48853	-0.25026	-2.8927
VAL250	-0.45394	-3.51764	-0.29802	-4.2696
GLY251	-0.09574	0.16565	-0.21073	-0.14082
PHE269	-0.0483	-0.38521	0.36238	-0.07112
ALA288	-0.88759	1.9543	-2.30391	-1.2372
ALA289	-1.88452	-1.23684	-1.41784	-4.53919
GLY290	-1.47742	-4.1642	1.82611	-3.81551
SER291	-0.38294	-0.13399	0.15503	-0.3619
ALA292	-0.1003	-0.82118	0.85611	-0.06536
VAL293	-0.77776	-1.53926	0.64649	-1.67053
SER294	-0.11048	-2.11151	1.46385	-0.75813
THR296	-0.03998	-0.61518	0.53943	-0.11574
ILE297	-0.31484	-0.74575	0.54648	-0.51411
PHE414	-0.03394	-0.0336	-0.02088	-0.08842
TRP426	-0.252	0.01483	-0.41775	-0.65492
PRO443	-0.03064	0.18204	-0.18663	-0.03523
TYR444	-0.05115	0.15613	-0.15567	-0.0507
HIS446	-0.06198	0.20636	-0.23791	-0.09352
ASN514	-0.62789	-1.68289	1.40965	-0.90112

TYR515	-2.11501	-7.02459	-0.64625	-9.78586
HIS516	-1.80129	-2.43908	0.18154	-4.05883
GLY517	-0.21422	0.55572	-0.90397	-0.56247
VAL518	-0.27245	-0.89384	0.4985	-0.66779
ILE547	-0.13642	0.24638	-0.38755	-0.27758
ASP548	-0.82708	25.67152	-24.65675	0.18768
GLY549	-0.48351	-6.09558	2.30047	-4.27862
SER550	-0.12202	-2.31435	1.32625	-1.11012
SER558	-0.13704	0.47595	-0.2795	0.05941
HIS559	-0.66673	-5.88537	-1.30392	-7.85602
VAL560	-1.35886	-0.43931	-1.98528	-3.78344
MET561	-0.5186	1.01392	-1.20221	-0.70689
THR562	-0.04353	0.49756	-0.54249	-0.08847
PHE564	-1.17763	-0.07397	-0.40918	-1.66078
MET567	-0.03946	-0.58702	0.46913	-0.15735
CS + FAD@GOx				
Residue	ΔE_{vdw}	ΔE_{ele}	ΔE_{sol}	ΔE_{total}
ILE24	-0.06469	-0.43107	0.42335	-0.07241
ALA25	-0.27575	0.66539	-0.94264	-0.553
GLY26	-0.73799	-2.01882	-0.22251	-2.97932
GLY27	-1.26313	-0.04785	-0.89186	-2.20284
GLY28	-1.32167	-7.72838	1.201	-7.84904
LEU29	-1.59293	-6.04709	1.84877	-5.79124
THR30	-0.20758	-7.80562	0.95465	-7.05854
GLY31	-0.11213	-2.51443	2.15589	-0.47067
ILE49	-0.4737	0.91493	-0.96935	-0.52812
GLU50	1.14717	8.55887	-23.67982	-13.97379
SER51	-1.29828	-1.60505	-0.48298	-3.3863
GLY52	-0.27579	-0.28456	0.54674	-0.0136
TYR68	-0.65628	-0.35525	-0.31998	-1.33151
GLY69	-0.02841	-0.16631	0.14558	-0.04914
ILE71	-0.03283	-0.08298	0.07972	-0.03609
PHE72	-0.31951	-0.18921	-0.16095	-0.66967
ASP77	-0.03617	9.9844	-9.87456	0.07367
HIS78	-0.1127	-0.90508	0.86209	-0.15569
TYR80	-0.09667	-0.22964	0.21611	-0.1102
ILE94	-0.12359	0.35506	-0.33351	-0.10204
ARG95	-0.66591	-8.04544	7.18952	-1.52183
SER96	-0.61566	-1.35403	0.53151	-1.43817
GLY97	-0.62604	1.25617	-1.34157	-0.71144
ASN98	-0.76923	-5.13233	0.89396	-5.0076
GLY99	-0.56392	-0.03858	-0.33113	-0.93363
LEU100	-0.61479	-0.69979	-0.37546	-1.69003
GLY101	-1.31406	-5.19319	1.20391	-5.30334
GLY102	-1.60563	-5.26058	1.40983	-5.45638
SER103	-1.57942	-18.75445	2.04724	-18.28663
THR104	-0.95644	-5.2686	2.35601	-3.86903

LEU105	-0.17876	-1.71701	1.7002	-0.19557
VAL106	-0.81403	-1.07713	0.49194	-1.39921
ASN107	-1.94853	0.65896	-0.99883	-2.2884
GLY108	-0.24995	-0.19904	0.0308	-0.41818
GLY109	-0.11771	-0.10273	0.24867	0.02823
THR110	-0.17295	0.54896	-0.45479	-0.07879
TRP111	-0.04708	0.00355	-0.06265	-0.10617
GLN248	-0.30084	0.38431	-0.52012	-0.43665
TYR249	-1.11751	-1.5685	-0.10029	-2.7863
VAL250	-0.51801	-3.09424	-0.18374	-3.79599
GLY251	-0.07422	0.23528	-0.24241	-0.08135
PHE269	-0.04552	-0.39372	0.36947	-0.06977
ALA288	-0.62297	1.76642	-2.00201	-0.85856
ALA289	-1.91735	0.18413	-2.04338	-3.7766
GLY290	-0.88881	-2.8995	1.6267	-2.16161
SER291	-0.25843	-0.26335	0.44208	-0.0797
VAL293	-0.75166	-1.74748	0.63741	-1.86173
SER294	-0.10893	-1.17853	0.94728	-0.34019
THR296	-0.039	-0.60267	0.54978	-0.09189
ILE297	-0.28513	-0.73184	0.58152	-0.43545
TYR300	-0.02342	-0.42991	0.42465	-0.02868
GLU412	-0.02622	3.61838	-3.72363	-0.13148
RPHE414	-0.02094	-0.0298	-0.00384	-0.05459
TRP426	-0.15509	-0.01182	-0.29188	-0.45879
PRO443	-0.03583	0.18585	-0.18955	-0.03953
TYR444	-0.05145	0.21145	-0.1992	-0.03921
HIS446	-0.08971	0.01748	-0.06789	-0.14013
ASN514	-0.63697	0.55857	-0.29177	-0.37017
TYR515	-2.213	-7.24536	-0.76167	-10.22002
HIS516	-1.69127	0.41497	-2.39041	-3.6667
GLY517	-0.1659	0.74286	-0.99886	-0.4219
VAL518	-0.11083	-0.53644	0.34055	-0.30673
ILE547	-0.07627	0.01695	-0.0397	-0.09902
ASP548	-0.54719	25.13773	-22.86831	1.72223
GLY549	-0.30902	-2.83348	2.19063	-0.95188
SER550	-0.07001	-1.59559	1.09986	-0.56574
SER558	-0.09711	0.67152	-0.41246	0.16195
HIS559	-0.28606	-7.67877	-0.72553	-8.69036
VAL560	-0.71595	-0.65846	-1.27095	-2.64536
MET561	-1.77475	0.51869	-1.70707	-2.96312
THR562	-0.0372	0.5107	-0.52985	-0.05634
PHE564	-1.14348	-0.24733	-0.60082	-1.99163
MET567	-0.05407	-0.89136	0.77003	-0.1754
PVA + FAD@GOx				
Residue	ΔE_{vdw}	ΔE_{ele}	ΔE_{sol}	ΔE_{total}
ILE24	-0.06733	-0.63111	0.5794	-0.11903
ALA25	-0.35064	0.65618	-0.90626	-0.60073

GLY26	-0.91997	-0.63716	-0.72275	-2.27988
GLY27	-1.00068	0.87809	-0.79737	-0.91997
GLY28	-1.57575	-7.79237	0.85915	-8.50897
LEU29	-2.24622	-8.3165	1.13469	-9.42803
THR30	0.09436	-14.00938	1.07963	-12.83539
GLY31	-0.18607	-2.94633	2.54676	-0.58563
ILE49	-0.43491	0.75115	-0.94377	-0.62752
GLU50	0.97442	9.24749	-22.93841	-12.7165
SER51	-1.51872	-0.78389	-0.14366	-2.44626
GLY52	-0.19963	-0.34197	0.51941	-0.02219
SER53	-0.03949	0.82576	-0.85818	-0.07191
TYR68	-0.33595	-0.44267	0.13639	-0.64224
GLY69	-0.01296	-0.10112	0.09336	-0.02073
ILE71	-0.06037	0.06963	-0.08753	-0.07826
PHE72	-0.36626	-0.1484	-0.3173	-0.83196
ASP77	-0.12605	11.34925	-11.28299	-0.05979
HIS78	-0.62367	-3.48444	1.02172	-3.08639
TYR80	-0.42362	-0.67026	0.57311	-0.52077
ILE94	-0.11086	0.62344	-0.5516	-0.03902
ARG95	-0.684	-8.96231	7.95828	-1.68803
SER96	-0.85922	-1.72196	0.31628	-2.2649
GLY97	-1.01887	1.08751	-1.86008	-1.79145
ASN98	-0.63962	-4.06328	0.89119	-3.81171
GLY99	-0.62783	3.1807	-1.93414	0.61873
LEU100	-0.2108	-0.48942	-0.19164	-0.89186
GLY101	-0.39234	0.06119	-0.01731	-0.34846
GLY102	-1.22567	-6.86686	0.36186	-7.73067
SER103	-1.33005	-16.90621	2.41523	-15.82104
THR104	-0.49454	-3.65138	2.47569	-1.67023
LEU105	-0.17136	-1.51563	1.74996	0.06298
VAL106	-0.90456	-1.10818	0.55312	-1.45962
ASN107	-1.83185	0.29501	-0.65991	-2.19676
GLY108	-0.27728	-0.32795	0.08896	-0.51627
GLY109	-0.10833	-0.24703	0.44325	0.08789
THR110	-0.69987	1.46235	-1.3619	-0.59942
TRP111	-0.0753	0.40187	-0.58175	-0.25519
THR112	-0.03756	0.22302	-0.26242	-0.07696
ALA228	-0.04746	-1.54841	1.29937	-0.2965
GLN248	-0.27061	0.52223	-0.60316	-0.35154
TYR249	-1.19746	-1.5827	-0.19725	-2.9774
VAL250	-0.45261	-3.28062	-0.25715	-3.99038
GLY251	-0.08083	0.15548	-0.17026	-0.09561
ALA288	-0.76475	1.5335	-2.3401	-1.57135
ALA289	-1.86436	-0.49258	-2.23839	-4.59533
GLY290	-1.17864	-3.51863	1.1945	-3.50276
SER291	-0.27615	-0.47112	0.58434	-0.16293
ALA292	-0.09631	-1.04235	1.07972	-0.05894

VAL293	-0.83437	-1.74036	0.62873	-1.946
THR296	-0.03986	-0.6876	0.61989	-0.10757
ILE297	-0.23894	-0.75478	0.61598	-0.37775
TYR300	-0.02977	-0.55927	0.55228	-0.03676
PHE414	-0.02904	-0.01974	-0.03002	-0.0788
TRP426	-0.20044	-0.04255	-0.38017	-0.62316
TYR444	-0.06482	0.19364	-0.19821	-0.06939
HIS446	-0.12517	0.08254	-0.1652	-0.20783
ASN514	-0.64816	0.11729	-0.05556	-0.58643
TYR515	-2.1922	-7.05646	-0.47644	-9.7251
HIS516	-1.98515	-4.26392	-0.7478	-6.99687
GLY517	-0.21718	0.62383	-1.03204	-0.62539
VAL518	-0.23021	-0.94203	0.55989	-0.61235
ILE547	-0.13085	0.03119	-0.20553	-0.30518
ASP548	-0.84597	25.51341	-24.2609	0.40654
GLY549	-0.39586	-6.75813	2.16898	-4.98501
SER550	-0.11714	-1.97032	1.14919	-0.93827
SER558	-0.08599	0.46087	-0.18742	0.18746
HIS559	-0.02059	-8.05631	-1.57444	-9.65135
VAL560	-0.51579	-0.52191	-1.24279	-2.28048
MET561	-2.13758	0.71174	-2.23967	-3.66551
THR562	-0.04456	0.50419	-0.5604	-0.10077
PHE564	-1.66343	0.30589	-0.79002	-2.14756
MET567	-0.0377	-1.24566	1.02157	-0.26179
Ce + CS + PVA + FAD@GOx				
Residue	ΔE_{vdw}	ΔE_{ele}	ΔE_{sol}	ΔE_{total}
ILE24	-0.07773	-0.62015	0.58915	-0.10874
ALA25	-0.47506	0.95578	-1.26114	-0.78042
GLY26	-1.12092	-0.39104	-1.10519	-2.61715
GLY27	-1.02457	0.96939	-0.86717	-0.92236
GLY28	-1.61377	-8.00871	0.71995	-8.90253
LEU29	-2.14131	-8.55892	0.76203	-9.93821
THR30	0.0644	-13.98531	0.88841	-13.0325
GLY31	-0.19527	-2.9919	2.48311	-0.70406
VAL48	-0.05835	-0.69355	0.62556	-0.12634
ILE49	-0.4479	0.74921	-0.91995	-0.61863
GLU50	0.9619	9.32262	-22.88139	-12.59688
SER51	-1.46184	-1.1594	-0.27043	-2.89167
GLY52	-0.20988	-0.37992	0.55406	-0.03574
TYR68	-0.6894	-0.297	-0.38453	-1.37094
GLY69	-0.03355	-0.16515	0.14499	-0.0537
ILE71	-0.05258	0.06414	-0.06683	-0.05527
PHE72	-0.44274	-0.1301	-0.40136	-0.97419
ASP77	-0.08258	10.99033	-10.89524	0.01251
HIS78	-0.62414	-3.63414	1.12891	-3.12937
TYR80	-0.41484	-0.61181	0.60392	-0.42273
ILE94	-0.10762	0.59248	-0.52964	-0.04479

ARG95	-0.60802	-8.73033	7.78584	-1.55251
SER96	-0.89144	-2.02974	0.62028	-2.3009
GLY97	-0.99426	1.17282	-1.7929	-1.61434
ASN98	-0.5435	-4.02978	0.71294	-3.86034
GLY99	-0.65553	3.02206	-1.88898	0.47755
LEU100	-0.20721	-0.50307	-0.06819	-0.77847
GLY101	-0.36741	-0.08093	-0.08943	-0.53777
GLY102	-1.21017	-6.96635	0.27734	-7.89919
SER103	-1.25852	-16.90029	2.43139	-15.72742
THR104	-0.45872	-3.61821	2.50226	-1.57467
LEU105	-0.16332	-1.5041	1.76063	0.09321
VAL106	-0.9387	-1.2048	0.60784	-1.53566
ASN107	-1.95258	0.74459	-1.15419	-2.36218
GLY108	-0.27323	0.10847	-0.33568	-0.50043
GLY109	-0.1087	0.15087	-0.00947	0.0327
THR110	-0.36361	0.5874	-0.63485	-0.41106
THR112	-0.03662	0.31066	-0.40263	-0.12859
ALA228	-0.04722	-1.47407	1.23083	-0.29045
GLN248	-0.29429	0.52579	-0.6242	-0.39271
TYR249	-1.09542	-1.66584	-0.04291	-2.80417
VAL250	-0.53314	-3.09004	-0.18927	-3.81245
GLY251	-0.07653	0.23824	-0.25591	-0.09419
LYS252	-0.01861	-7.9526	7.84503	-0.12618
PHE269	-0.04413	-0.37689	0.35413	-0.0669
ALA288	-0.8468	1.73179	-2.55382	-1.66883
ALA289	-1.86227	-0.83768	-1.83638	-4.53633
GLY290	-1.26401	-3.75269	1.40046	-3.61624
SER291	-0.30903	-0.69722	0.65114	-0.35511
ALA292	-0.0935	-0.97091	1.0212	-0.0432
VAL293	-0.73829	-1.71124	0.60424	-1.84529
THR296	-0.04011	-0.67207	0.60218	-0.11001
ILE297	-0.29229	-0.76981	0.6372	-0.42491
TYR300	-0.02169	-0.32023	0.31752	-0.0244
PHE414	-0.02539	-0.0216	-0.02384	-0.07082
TRP426	-0.20548	0.02791	-0.37927	-0.55684
PRO443	-0.02415	0.20318	-0.20724	-0.02822
TYR444	-0.04984	0.09158	-0.09509	-0.05334
HIS446	-0.10922	-0.18111	0.13517	-0.15516
ASN514	-0.60293	0.07242	0.14729	-0.38322
TYR515	-1.97103	-7.13703	-0.28991	-9.39797
HIS516	-2.09662	-5.66063	-0.97156	-8.72881
GLY517	-0.23382	0.77074	-1.16217	-0.62525
VAL518	-0.2224	-0.91716	0.58927	-0.55029
ILE547	-0.12481	0.15395	-0.27762	-0.24848
ASP548	-0.82397	25.88905	-24.31257	0.75251
GLY549	-0.40294	-6.43545	2.2995	-4.53889
SER550	-0.11057	-1.96226	1.19221	-0.88062

SER558	-0.09484	0.48736	-0.2227	0.16982
HIS559	-0.13997	-8.0429	-1.11797	-9.30084
VAL560	-0.46137	-0.59988	-1.18492	-2.24617
MET561	-0.59411	0.99383	-1.33312	-0.9334
THR562	-0.04254	0.68839	-0.72356	-0.07771
PHE564	-1.5365	-0.49223	-0.35949	-2.38821
MET567	-0.06174	-0.98905	0.83812	-0.21267
